# Chrysosplenetin acts as a homeostasis stabilizer with dual-function in shattering *Plasmodium berghei* K173 resistance to artemisinin driven by both ABC transporters and heme-ROS/GSH axis

**DOI:** 10.1186/s13071-025-07018-0

**Published:** 2025-10-09

**Authors:** Hongyan Ji, Yutao Huang, Jingxuan Tian, Ruonan Bo, Xin Heng, Junyi Yang, Yifan Huang, Xiangyu Wang, Qingfeng Tan, Hua Gao, Linwei Chen, Jing Chen

**Affiliations:** 1 Institute of Clinical Pharmacology, General Hospital of Ningxia Medical University, Yinchuan, 750004 Ningxia People’s Republic of China; 2 School of Traditional Chinese Medicine, Faculty of Medicine, Yangzhou University, Yangzhou, 225009 People’s Republic of China; 3 College of Veterinary Medicine, Jiangsu Co-Innovation Center for Prevention and Control of Important Animal Infectious Diseases and Zoonoses, Yangzhou University, Yangzhou, 225009 Jiangsu People’s Republic of China; 4 Department of Pharmacy, The Affiliated Taizhou People’s Hospital of Nanjing Medical University, Taizhou, 225300 Jiangsu People’s Republic of China; 5 The Key Laboratory of the Jiangsu Higher Education Institutions for Nucleic Acid & Cell Fate Regulation (Yangzhou University), Yangzhou, 225001 People’s Republic of China; 6 The Key Laboratory of the Jiangsu Higher Education Institutions for Integrated Traditional Chinese and Western Medicine in Senile Diseases Control (Yangzhou University), Yangzhou, 225001 People’s Republic of China

**Keywords:** Chrysosplenetin, Artemisinin resistance, ABC transporters, Cytokines, PI3K/AKT-mTOR and MAPK pathways, Heme-ROS/GSH axis

## Abstract

**Background:**

Chrysosplenetin (CHR), a polymethoxy flavonol co-occurring with artemisinin (ART) in *Artemisia annua* L., reverses ART resistance in *Plasmodium berghei* K173 potentially by downregulating intestinal P-glycoprotein (P-gp, encoded by *Mdr1a*) expression. In the present study, we further elaborated on the mechanism by comparing differences in antimalarial activity and resistance-associated molecular expression profiles between ART alone and combination therapy in blood and tissues of *Mdr1a* wild-type (WT) and knockout (KO) mice infected with either sensitive or resistant malarial parasites.

**Methods:**

We evaluated the effects of monotherapy and combination therapy in WT and KO mice infected with sensitive and resistant *P. berghei* K173 strains. The mRNA expressions of multi-resistance proteins (Mrp1, 2, 4, 5) and breast cancer resistance proteins (Bcrp) were detected. Hemoglobin levels, mRNA expressions of cytokines including tumor necrosis factor-α (IFN-α), interferon-α (IFN-α), and interleukin (IL-1β) in blood and tissues, and redox balance (ROS/GSH levels), as well as gene or protein expression of signaling pathway (PI3K/AKT-mTOR and MAPK) were investigated.

**Results:**

In drug-resistant mice, combination therapy maintained the highest survival (100%) and inhibition (30%) rates and the lowest parasitaemia percentage (approximately 20.0%), irrespective of *Mdr1a* gene status. Furthermore, combination reshaped the spatial and ART resistance-phenotypic disparities in Mrps and Bcrp mRNA expressions (with a fold change ranging from 1.35 to 38.03), ROS/GSH balance (ranging from 1.02-fold to 10.18-fold), hemoglobin levels (ranging from 1.04-fold to 1.20-fold), and cytokine profiles (ranging from 1.14-fold to 37.79-fold) induced by ART alone, which were partially dysregulated by *Mdr1a* deficiency. Monotherapy and combination exert oppositely regulatory effects on the PI3K/AKT-mTOR pathway in a tissue-, *Mdr1a* genotype-, and parasite sensitivity/resistance-dependent manner (ranging from 1.52-fold to 84.00-fold). Specifically, CHR reversed ART-induced changes via PI3K/AKT protein inhibition (ranging from 1.20-fold to 63.00-fold), which was contingent on P-gp functionality. Finally, mitogen-activated protein kinase (MAPK) pathway was involved in the antagonistic regulation between ART alone and combination therapy in a P-gp-independent manner (ranging from 1.39-fold to 16.69-fold).

**Conclusions:**

The efflux pump function of P-gp is probably not a critical factor in the mechanism by which CHR reverses ART resistance. Instead, CHR acts as a homeostasis stabilizer with dual functions: it disrupts *Plasmodium berghei* K173 resistance to ART driven by both ABC transporters and the heme-ROS/GSH axis, in which the non-transport function of P-gp on ART is involved.

**Graphical Abstract:**

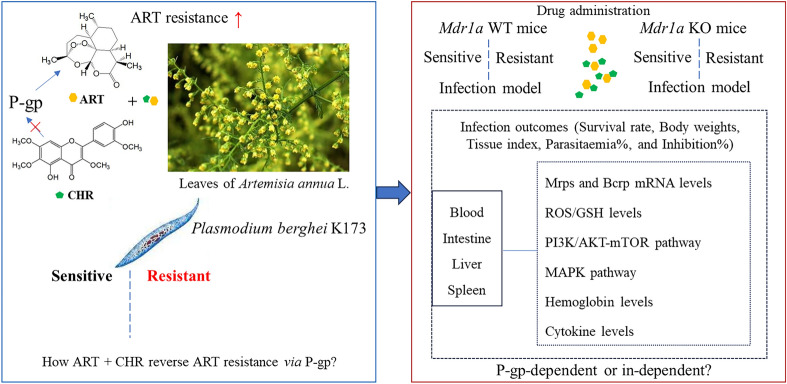

**Supplementary Information:**

The online version contains supplementary material available at 10.1186/s13071-025-07018-0.

## Background

Malaria, one of the most lethal parasitic diseases, has posed a serious threat to human health for more than 1000 years [1]. Globally, an estimated 249 million malaria cases and 608,000 malaria-related deaths were reported across 85 countries in 2022 [2]. To date, artemisinin (ART) and its derivatives in combination therapies (ART-based combination therapies, ACTs) remain recommended by the World Health Organization as first-line antimalarial drugs, with potential therapeutic applications extending beyond their antimalarial effects [3]. However, over the past decade, partial ART resistance has emerged in the Greater Mekong subregion, serving as a warning sign that could undermine global efforts to control malaria [4]. Regrettably, the multiple mechanisms underlying ART resistance are extremely complex and remain largely undefined.

Parasites obtain amino acids required for their growth and development within the host by digesting hemoglobin in the food vacuole (FV). Concurrently, the toxic by-product free heme is converted into non-toxic hemozoin (Hz) crystal in the FV via hydrogen bonding between the carboxy side chains of the protoporphyrin ring [5]. Currently, it is well established that heme is essential for the specific activation of ART. Upon binding to heme iron, the peroxide bridge of ART is cleaved [6, 7], generating oxygen- or carbon-centered free radicals and reactive oxygen species (ROS). These ROS can alkylate various crucial proteins of *Plasmodium*, particularly cysteine proteases that facilitate hemoglobin uptake by the parasite, thereby causing these essential proteins to lose their original functions. Subsequently, the antioxidant glutathione (GSH) system is activated to regulate ROS levels. GSH, a tripeptide consisting of cysteine, glycine, and glutamate, plays a vital role in the defense system [8]. As one of the free-radical scavengers, it effectively eliminates excessive ROS or endogenous and exogenous electrophilic substances to maintain intracellular redox homeostasis.

ATP-binding cassette (ABC) transporters are a family of transmembrane transport proteins. Consisting of 48 members across 7 subfamilies (A-G) [9], they are widely distributed in diverse organisms [10]. These transporters mediate the transport of numerous substances, including amino acids, peptides, proteins, sugars, heme, cellular metabolites, and drugs, by utilizing energy generated from ATP hydrolysis [10, 11]. Additionally, ABC transporters are involved in maintaining cholesterol homeostasis, regulating oxidative stress responses, supporting tissue defense, mediating immune recognition, and performing various other physiological functions [12]. Abnormal expressions of ABC transporters in cells can influence drug absorption, distribution, metabolism, and excretion, thereby contributing to multidrug resistance (MDR) [13]. Key members of this family include P-glycoprotein (P-gp), MDR-associated proteins (rodent Mrps or human MRPs, the same applies below), and breast cancer resistance relative protein (Bcrp/BCRP). P-gp, encoded by the *ABCB1* (*Mdr1*/*MDR1*) gene, reduces intracellular drug accumulation by pumping drugs out of cells. P-gp-mediated MDR is one of the most prominent intrinsic mechanisms underlying ART resistance [14]. Mrps, encoded by the *ABCC* family, alter the pH of organelles and the cytoplasm. They utilize energy from ATP hydrolysis to transport negatively charged drugs against the concentration gradient [15]. Substrates of the Mrps family include lipophilic compounds such as GSH and glucuronic acid [16]. Bcrp, encoded by the *ABCG2* gene, mitigates hypoxic conditions induced by heme porphyrin and their metabolites [17] and enhances cellular antioxidant capacity, maintaining the dynamic balance of ROS [18]. Certain inflammatory factors, such as TNF-*α* and interleukin (IL)-1*β*, can directly inhibit Bcrp expression [19]. Excessive ABC transporter expression is typically regulated by the PI3K/AKT-mTOR signaling pathway, the mitogen-activated protein kinase (MAPK) signaling pathway, and the levels of specific inflammatory factors [20, 21]. PI3K inhibitors (LY294002 or PI-103) sensitize the colorectal cancer cells to the chemotherapeutic drug 5-fluorouracil by reducing the expression of ABCG2 and ABCG5 [22], while the mTOR-specific inhibitor rapamycin exerts differential effects on the levels of ABC transporter proteins and regulates these transporters at the level of translation [23]. However, whether P-gp, in turn, affects these pathways or cytokines remains unknown.

Our previous study revealed that CHR, a polymethoxylated flavone extracted from the acetone fraction of ART industrial waste, exerted a direct parasiticidal effect and simultaneously sensitized ART-resistant *Plasmodium berghei* K173, with no impact on sensitive strains [24]. Furthermore, when combined with ART at a 2:1 ratio, it downregulated P-gp expression in the small intestine, inhibited in vivo P-gp transport activity, and modulated differences in heme/Hz balance differences between mice infected with sensitive and resistant parasites [24]. In the present study, we further explored the interaction network between MDR-mediated cross-resistance and heme-ROS/GSH-triggered specific resistance to ART (both in the absence and presence of CHR), as well as the underlying molecular mechanisms using a *Mdr1a*-KO mouse model.

## Methods

### Chemicals and reagents

ART (batch no. 190711Q, with a purity of more than 98%) was purchased from Aladdin Reagent Co., Ltd. (Shanghai, China). CHR (with a purity of more than 95%) was purified in our laboratory and its chemical structure was authenticated by analysis of proton nuclear magnetic resonance (^1^H NMR) and carbon-13 nuclear magnetic resonance (^13^C NMR) [25]. Sodium carboxymethylcellulose (CMC-Na), sodium citrate (99.0%), sodium chloride (batch no. 20211028), potassium dihydrogen phosphate (99.5%), trisodium phosphate (99.0%), methanol, and absolute ethyl alcohol were supplied by Sinopharm Chemical Reagent Co., Ltd. (Shanghai, China).

Rifampicin (RIF, with a purity of more than 97%), a mouse tail genotype rapid identification kit, agarose, nucleic acid (NA)-red dye (batch no. 051421220117), 50× tris–acetate-ethylenediaminetetraacetic acid (EDTA) electrophoresis buffer (TAE), Easy-Load^™^ PCR Master Mix (Green, 2×), phosphate buffered saline (PBS, 10× , premixed powder), diethylpyrocarbonate (DEPC)-treated water, Trizol, DNA loading buffer, a reactive oxygen species (ROS) detection kit, a bicinchoninic acid assay (BCA) protein concentration assay kit, a total glutathione (GSH) assay kit, radio-immunoprecipitation assay (RIPA) lysate (medium-strength), deionized water, protein loading buffer, skim milk powder, polyvinylidene fluoride (PVDF) film, a *β*-actin mouse monoclonal antibody, primary antibody dilution buffer, and a DNA ladder were all provided by Beyotime Biotechnology Co., Ltd. (Shanghai, China). A rapid Wright stain solution and an enhanced chemiluminescence (ECL) detection kit were purchased from Jiangsu KeyGEN BioTECH Co., Ltd. (Nanjing, China). Gene primers were obtained from Beijing Robbie Biotechnology Co., Ltd. (Beijing, China). The primer sequences of internal reference gene and the targeted genes are presented in Table 1.

**Table 1:** Primer sequences of internal reference genes and encoding genes of ABC transporters and cytokines, as well as those involved in the PI3K/AKT-mTOR and MAPK pathways

Gene	Primer (5'-3')	Annealing temperature (℃)	Fragment length (bp)
GAPDH	GGTGAAGGTCGGTGTGAACG	55.9	20
	CTCGCTCCTGGAAGATGGT	53.2	19
Mrp1	TTCGGAAGGGAGAATCGGCTTCAA	57.4	24
	TGTTCAGTGCACCTTGCTTGTTCG	57.4	24
Mrp2	TCCAGGACCAAGAGATTTGC	51.8	20
	TCTGTGAGTGCAAGAGACAGGT	54.8	22
Mrp4	TAATGGAAGCAGACAAGGCCCAGA	57.4	24
	AGAGGCCAGTGCAGATACATGGTT	57.4	24
Mrp5	ACAGCCGCTATGGACACAGAGACAGA	61.1	26
	AGGCGAAGTTTCAGCAGGACAGGATG	61.1	26
Bcrp	GCATTCGCTGTGGTTGAGT	51.1	19
	TATCCGTGGCATCTCTGGA	51.1	19
PI3K	ACACCACGGTTTGGACTATGG	54.4	21
	GGCTACAGTAGTGGGCTTGG	55.9	20
AKT	ATGAACGACGTAGCCATTGTG	52.4	21
	TTGTAGCCAATAAAGGTGCCAT	51.1	22
m-TOR	CAGTTCGCCAGTGGACTGAAG	56.3	21
	GCTGGTCATACAAGCGAGTAGAC	57.1	23
ERK	GGTTGTTCCCAAATGCTGACT	52.4	21
	CAACTTCAATCCTCTTGTGAGGG	55.5	23
JNK	AGCAGAAGCAAACGTGACAAC	52.4	21
	GCTGCACACACTATTCCTTGAG	55.4	22
P38	TGACCCTTATGACCAGTCCTTT	53.6	22
	GTCAGGCTCTTCCACTCATCTAT	55.5	23
TNF-*α*	CAGGCGGTGCCTATGTCTC	55.8	19
	CGATCACCCCGAAGTTCAGTAG	57.2	22
IFN-*γ*	GCCACGGCACAGTCATTGA	53.6	19
	TGCTGATGGCCTGATTGTCTT	52.4	21
IL-1*β*	GAAATGCCACCTTTTGACAGTG	53.6	22
	TGGATGCTCTCATCAGGACAG	52.4	21

HiScript Ⅲ RT SuperMix for quantitative polymerase chain reaction (qPCR) (+ gDNA wiper) and ChamQ Universal SYBR qPCR Master Mix were purchased from Nanjing Vazyme Biotech Co., Ltd. (Nanjing, China). Glycine (batch no. 923W069, with a purity of more than 99.0%), tris–HCl (1.0 M, pH = 6.8), tris–HCl (1.5 M, pH = 8.8), and tris were acquired from Beijing Solarbio Science & Technology Co., Ltd. (Beijing, China). The rabbit monoclonal anti-PI3 kinase p85*α* antibody (clone number: EPR18702; catalog number: ab191606; dilution: 1/10000) and anti-AKT1 + AKT2 + AKT3 antibody (clone number: EPR16798; catalog number: ab179463; dilution: 1/1000) were sourced from Abcam Inc. USA. Anti-beta action (catalog number: ab8227; dilution: 1/10000) polyclonal antibody was also purchased from Abcam Inc. (USA). Horseradish peroxidase (HRP)-conjugated goat anti-mouse IgG (catalog number: A21020; dilution: 1/10000) was purchased from Abbkine Inc. (USA). Ammonium persulfate (with a purity of more than 98%) and N,N,N′,N′-tetramethylethylenediamine (TEMED) were sourced from Sigma Corporation (USA). Tween 20 and sodium dodecyl sulfate were provided by Biotopped Corporation (USA).

### Malaria parasite strains

As reported in our previous study [24], ART-sensitive and ART-resistant strains of *Plasmodium berghei* K173 were kindly provided by Professor Yong Dai from the Research and Experimental Center of the Basic Medical College at Chengdu University of Traditional Chinese Medicine. The resistant strain was cultivated up to 42 generations using a method of equivalent increment, resulting in a resistance index of 14.153 [24]. Prior to use, the parasite strains were stored in a refrigerator at −80 ℃.

### Experimental animals

The WT genotype male Institute of Cancer Research (ICR) mice, which weighed 18.0–22.0 g at baseline before infection, were obtained from the Comparative Medicine Center of the College of Veterinary Medicine at Yangzhou University (SYXK-Su-2022–0044). The breeding strategy for *Abcb1a* KO inbred mice on Friend Virus B-type (FVB) background supplied by Nanjing Model Organisms is detailed below. Heterozygous × heterozygous crosses (*Abcb1a*^+^/^−^ × *Abcb1a*^+^/^−^) are performed to yield offspring with a genotypic distribution of 25% *Abcb1a*^−^/^−^ (homozygous KO), 50% *Abcb1a*^+^/^−^ (heterozygous), and 25% *Abcb1a*^+^/^+^ (WT). For genotyping purposes, DNA is extracted from tail tissues at 3–4 weeks of age, followed by PCR-based analysis using supplier-provided primers and sequencing to validate the targeted exon deletion. Females at 6–8 weeks of age and males at 8–10 weeks of age were mated in a 1:2–3 ratio, with pups weaned at 21 days. The average litter size was 6–8 offspring. For strain maintenance, heterozygous (*Abcb1a*^+^/^−^) mice were retained as breeders, while viable *Abcb1a*^−^/^−^ homozygotes weighing 18–22 g were utilized for experimental purposes or further breeding.

The animals were housed in cages with free access to standard laboratory food and water in a specific-pathogen-free (SPF) environment, maintained at a relative humidity of 50–70%, room temperature of 24.0 ± 2.0 ℃, and 12-h light–dark cycle. They were allowed a 1-week acclimation period prior to the initiation of any experimental procedures. All animal-related protocols were submitted to and approved by the University Ethics Committee (YXYLL-2021-13). The animals were handled in accordance with the Regulations of the Experimental Animal Administration issued by the State Committee of Science and Technology.

### Genotype identification of KO mice using a mouse tail-based validation method

#### Genomic DNA extraction from mouse tails

Approximately 0.2–1.0 cm of the mouse tail tip was cut using a pair of scissors that had been rinsed with 75% ethanol. The cut tail tip was then completely immersed in 100 μL of digestive solution. The samples were placed in a water bath set at 55 ℃ and incubated for 15 min. Subsequently, the samples were transferred to a 95 ℃ water bath and incubated repeatedly for 5-min intervals. After that, 100 μL of stop solution was added to the samples, which were then vortexed thoroughly. The extracted DNA extractions were stored at −20 ℃ for long-term preservation and at 4 ℃ for short-term use.

#### PCR amplification

Thawed biosamples and PCR reagents were kept on ice and mixed to prepare a 20-μL reaction system containing 7.4 μL of Milli-Q water, 1 μL of template (2–20 ng/μL), 0.8 μL of each primer (10 μM stock, final 0.8 μM), and 10 μL of Easy-Load™ PCR Master Mix (Green, 2×). The reaction was run on a Bio-Rad T100^™^ Thermal Cycler with gradient functionality for PCR optimization (Bio-Rad Co., Ltd., German) with parameters: initial denaturation at 94 °C for 3 min, followed by 30–35 cycles of 94 °C for 30 s, 55 °C for 30 s, and 72 °C for 1 min/kilobase (kb), with a final extension at 72 °C for 10 min and a 4 °C hold. PCR products were stored at −20 °C.

#### Agarose gel electrophoresis

To prepare the agarose gel, 100 mL of electrophoresis buffer was added to a conical flask, followed by 1.0 g of agarose, which was then dissolved using a microwave oven. After cooling the mixture to around 60 °C, 5 μL of NA-red dye was added to create a 1.0% agarose gel. The gel was poured into a 10 cm × 10 cm gel plate with a 1.5 mm comb and left to solidify. The solidified gel was placed in a NA-Gel^™^ Nucleic Acid Electrophoresis System (Beyotime Institute of Biotechnology) filled with electrophoresis buffer. Samples (5 μL each) were loaded, and electrophoresis was performed at 120 V for 35 min. After the run, the gel was transferred to an ultra-sensitive automated imaging analysis system (Leica Clara, USA) for visualization of the electrophoretic bands. As shown in Supplementary Material Fig. 1, PCR amplification of the target gene region in KO mice yielded a single 387-base pair (bp) product (homozygous KO mice, *Mdr1a* −/−), whereas WT mice exhibited a single 418-bp fragment (homozygous WT mice, * Mdr1a* +/+). Although sequencing was not performed in this study, the distinct 31-bp size difference between WT and KO amplicons directly reflects the targeted deletion of a 31-bp exon in the KO genome; combined with strict control measures, this provides robust confirmation of the KO status.

### Recovery, passage, and infection of ART-sensitive and ART-resistant parasites in WT and KO mice

The detailed experimental procedures were reported in our previous work [24]. A total of 36 or 48 healthy male ICR mice (18.0–22.0 g) were randomly assigned to WT and KO subgroups. Each subgroup was infected with either sensitive or resistant parasites (6–8 mice per group). The mice were intraperitoneally injected with parasite-infected blood at a dose of 1 × 10^5^ red blood cells (RBCs) per mouse. Tail vein blood samples were collected for smears, and Wright’s stain was used to determine the infection rate. Microscopic analysis showed that *P. berghei* infection induces morphological alterations in RBCs, including membrane deformation and cytoskeletal disruption (Supplementary Material Fig. 2). Wright’s staining clearly visualized the parasites, with the majority in the trophozoite stage (characterized by a ring shape in the early phase and amoeboid irregularity in the late phase and the schizont stage (defined by nuclear division in the early phase and formation of distinct merozoites in the mature phase). Quantitative analysis of these slides yielded data on parasitaemia and inhibition rates. Mice with an infection rate of 20% were designated as passage parent mice. Blood (9–12 drops) was collected from these mice via retro-orbital puncture and added into a preservation solution containing 10 mL normal saline and 0.05 mL sodium citrate. This solution was then intraperitoneally injected into other healthy mice at 1 × 10^5^ RBCs per mouse. Once the infection rate exceeded 5%, the mice were weighed to record their initial body weights (day 1; approximately 23–32 g) and utilized for subsequent experiments. Sterile techniques were strictly maintained in a biological safety cabinet (Thermo Fisher Scientific, USA) throughout the experiment.

### Drug administration and collection of blood and tissues

The drug administration groups consisted of control subgroup (0.5% CMC-Na, 13 mL/kg), ART alone subgroup (ART, 40 mg/kg), and combination subgroup (ART:CHR = 1:2, 40:80 mg/kg). This ratio between ART and CHR was optimized in our laboratory [24]. RIF (210 mg/kg) was used as a classic P-gp inducer to KO mice, which helps distinguish whether the combination’s resistance-reversing effects are P-gp-dependent or independent. Therefore, the overall animal grouping is as follows: WT-sensitive group (CMC-Na, monotherapy, and combination subgroup), WT-resistant group (CMC-Na, monotherapy, and combination subgroup), KO-sensitive group (CMC-Na, monotherapy, combination, and RIF subgroup), and KO-resistant group (CMC-Na, monotherapy, combination, and RIF subgroup). All drugs were suspended in a 0.5% CMC-Na solution. The infected mice were administered the abovementioned drugs once a day for seven consecutive days, and 1 h after each administration, blood samples were collected to monitor the infection rate. Body weights were recorded at the same timepoint each day. On day 7, peripheral blood was collected by eyeball enucleation. After euthanasia, the mice were dissected, and the intestinal, splenic, and hepatic organs were harvested. These organs were washed three times with PBS buffer, and excess water was blotted out. The weight of each organ was recorded, placed in a frozen storage tube, and then stored in a refrigerator at −80 ℃ until use.

### Differences in antimalarial activity between ART alone and combination therapy within WT and KO mice infected with sensitive and resistant parasites

Malarial infection rates, inhibition rates, body weights, survival curves, and organ indexes were detected. The infection rate (parasitaemia%, denoted as *Y*_*1*_, Eq. 1) and inhibition rate (denoted by *Y*_*2*_, Eq. 2) were calculated using the following formulas:

1 $$Y_{1} \, = \,\frac{{\text{Total number of infected RBCs}}}{{\text{Total number of RBCs}}}\, \times \,100\%$$

2 $$Y_{2} \, = \,\frac{{{\text{Infection rate in the control groups}} - {\text{Infection rate in the drug administration groups}}}}{{\text{Infection rate in the control groups}}}\, \times \,100\%$$

Four consecutive fields were randomly selected under a high-power microscope as statistical samples. The total number of RBCs within each visual field and the total number of RBCs infected by *Plasmodium* were counted separately.

### ROS and GSH detection in blood and tissues of WT and KO mice infected with sensitive and resistant parasites under ART alone and combination therapy

Fresh blood samples were immediately transferred to precooled (4 ℃), enzyme-free 1.5 mL Eppendorf centrifuge tubes, and placed on ice for 15 min. Samples were then centrifuged at 600 × *g* for 10 min at 4 ℃. The supernate (plasma) was carefully transferred to a new precooled, enzyme-free centrifuge tube. For tissue samples, 50 mg each of intestine, spleen, and liver were separately placed into pre-cooled enzyme-free centrifuge tubes. These tissues were washed twice with ice-cold PBS buffer, centrifuging at 1000 × *g* for 5 min at 4 ℃ in a bench-top low-temperature high-speed centrifuge (Dalong Xingchuang Experimental Instrument Co., Ltd., China) between washes. The relative content of ROS in blood and tissue samples was measured using a commercial ROS detection kit. Fluorescence readings were obtained with an EnSpire Multimode Microplate Reader (PerkinElmer, Singapore) set at an excitation wavelength of 488 nm and an emission wavelength of 526 nm. GSH levels were determined using a commercially available assay kit. After a 25-min incubation period, the absorbance of standards and samples was measured at 412 nm with a microplate reader (BioTek, USA).

### Measurement of of the major ABC transporter mRNA levels by real-time quantitative PCR (RT-qPCR)

RNA reverse transcription was carried out following the instructions of the HiScript Ⅲ RT SuperMix for qPCR (+ gDNA wiper) kit. The resulting reaction products could either be immediately used for the qPCR reaction or stored at −20 ℃ until needed. The primer sequences for several key ABC transporter genes, namely Mrp1, Mrp2, Mrp4, Mrp5, and Bcrp, along with that of the housekeeping gene GAPDH, were presented in Table 1. The qPCR experiment was conducted using the ChamQ Universal SYBR qPCR Master Mix kit. The qPCR reaction system consisted of 10.0 μL of 2 × ChamQ Universal SYBR qPCR Master Mix, 0.4 μL of forward primer (10 μM), 0.4 μL of reverse primer (10 μM), 2.0 of complementary DNA (cDNA), and 7.2 μL of ddH_2_O with a total volume of 20.0 μL. The qPCR reaction conditions were as follows: stage 1 (denaturation stage), 1 cycle at 95 ℃ for 30 s; stage 2 (cycling stage), 40 cycles, with each cycle consisting of 95 ℃ for 10 s and 60 ℃ for 30 s; and stage 3, melt curve stage, 1 cycle, involving 95 ℃ for 15 s, 60 ℃ for 30 s, and 95 ℃ for 15 s.

After the completion of the RT-qPCR reaction, cycle threshold (Ct) value of each sample was obtained. The 2^−△△Ct^ relative quantitative method was employed to analyze the expressions of related genes in blood and different tissue samples.

### Measurement of mRNA or protein levels in phosphoinositide 3-kinase-protein kinase B/mammalian target of rapamycin (PI3K/AKT-mTOR) and MAPK signaling pathways

The qPCR gene primer sequences for the target proteins are presented in Table 1. The experiments were conducted using the same procedures as described above.

The western blot technique was utilized to detect the relative expression levels of PI3K and AKT in the small intestines. A total of 30 mg of intestinal tissues were lysed in RIPA buffer containing 1% EDTA, 1% phosphatase inhibitors, and 1% phenylmethylsulfonyl fluoride (PMSF) to preserve protein integrity, followed by high-speed centrifugation (12,000 × *g*, 4 ℃ for 15 min) to remove debris and quantification of total protein using a BCA assay. Next, 20–30 μg of protein samples were loaded onto a 10–12% polyacrylamide gel for sodium dodecyl sulfate–polyacrylamide gel electrophoresis (SDS-PAGE) electrophoresis under an electric field. After electrophoresis, proteins were electrotransferred to a PVDF membrane. The membrane is blocked with 5% non-fat milk in tris-buffered saline with Tween 20 (TBST) for 1 h at room temperature, then incubated overnight at 4 °C with primary antibodies against PI3K, AKT, and a loading control (*β*-actin). Following washing with TBST, the membrane was incubated with HRP-conjugated secondary antibodies for 1 h and washed again. Signals were detected using an ECL substrate and chemiluminescence imaging system. The intensity of the bands was quantified through densitometry analysis with ImageJ version 2 × (NIH Image software, Bethesda, MA, USA).

### Detection of TNF-α, IFN-γ, and IL-1β mRNA levels by RT-qPCR

The gene primer sequences for TNF-*α*, IFN-*γ*, and IL-1*β* are presented in Table 1. The RT-qPCR experiments were performed using the same methods as previously described.

### Statistical analysis

All data were presented as $\overline{x}$ ± standard deviation (SD). The sample size was *n* = 6–8 or 3–4 depending on experimental requirements. Survival curves were estimated using the Kaplan–Meier method and statistically analyzed with log-rank (Mantel–Cox) test, employing Graphpad Prism 8.0 (USA). For other data, SPSS 18.0 software (IBM, USA) or Graphpad Prism 8.0 was used. The data were subjected to one-way analysis of variance (ANOVA) to detect significant differences among study groups. The Student–Newman–Keuls (SNK) was applied to determine difference between means with significance levels set at* P* < 0.05, *P* < 0.01, and *P* < 0.001.

## Results

### ART–CHR combination therapy improves infection outcomes in ART-resistant *Plasmodium*-infected mice associated with P-gp status

**Fig 1.**
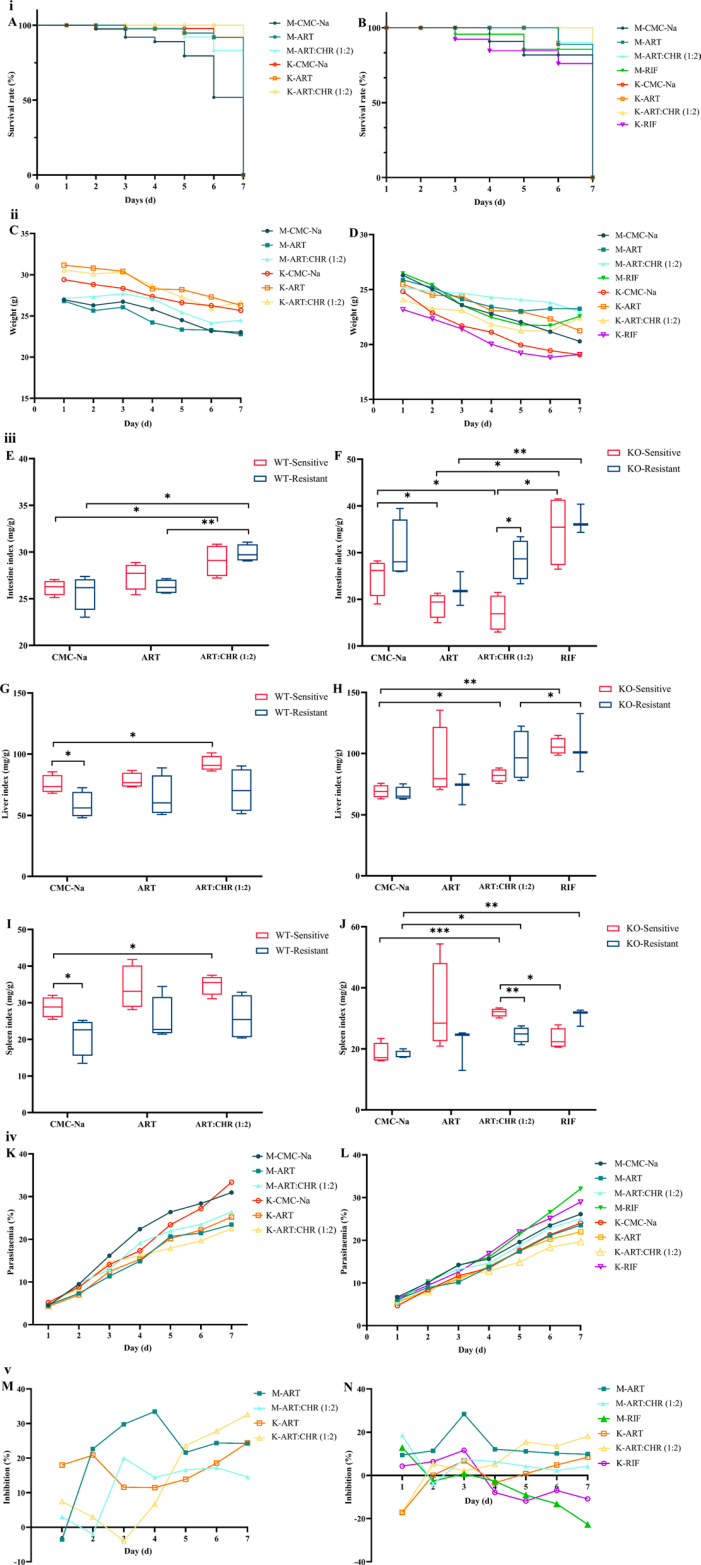
Opposing regulatory effects between monotherapy and combination on infection outcomes over 7 days and ART’s antimalarial activity in sensitive and resistant mice, operating via either a P-gp-dependent or P-gp-independent mechanism. All data were presented as $\overline{x}$ ± standard deviation (SD, *n* = 6–8). The data were subjected to one-way analysis of variance (ANOVA) to detect significant differences among study groups. The Student–Newman–Keuls (SNK) was applied to determine difference between means, with significance levels set at* P* < 0.05, *P* < 0.01, and *P* < 0.001. **i** Survival rate (%); **ii** body weights (g); **iii** organ indexes (mg/g); **iv** parasitaemia percentage (%); and **v** inhibition percentage (%). **A** Survival rate in WT mice;** B** survival rate in KO mice; **C** body weights in WT mice; **D** body weights in KO mice; **E** intestine index in WT mice; **F** intestine index in KO mice; **G** liver index in WT mice; **H** liver index in KO mice; **I** spleen index in WT mice; **J** spleen index in KO mice; **K** parasitaemia percentage in WT mice; **L** parasitaemia percentage in KO mice; **M** inhibition percentage in WT mice; and **N** inhibition percentage in KO mice. CMC-Na: carboxymethyl cellulose sodium, 13 mL/kg; ART: monotherapy, 40 mg/kg; ART–CHR (1:2), artemisinin–chysosplenetin combination in ratio of 1:2, 40:80 mg/kg; RIF: rifampicin, 210 mg/kg. M–CMC–Na, M–ART, and M–ART–CHR (1:2) represent the CMC-Na, ART, and combination treatments in sensitive WT or KO mice, respectively (*n* = 6–8). K–CMC–Na, K–ART, and K–ART–CHR represent the CMC-Na, ART, and combination treatments in resistant WT or KO mice, respectively (*n* = 6–8). M-RIF and K-RIF represent the RIF treatment in sensitive and resistant KO mice, respectively (*n* = 6–8)

As illustrated in Fig. 1i A,B, during the 7-day drug-treatment course, KO-sensitive mice receiving CMC-Na treatment (M-CMC-Na) exhibited a relatively higher survival rate (80.2%) compared with their WT counterparts (50%). Combination therapy substantially augmented the survival rate (100%) in drug-resistant mice, irrespective of *Mdr1a* gene status (Fig. 1iA, B). Notably, among all experimental cohorts, RIF caused the most significant reduction in survival rate (75%) in the KO-resistant group (presumed to lack functional P-gp) (Fig. 1i B). Taken together, these findings suggest that P-gp may exacerbate infection-associated harm, with its deficiency conferring protective benefits to the host. Additionally, in the resistant *Plasmodium* infection model, the therapeutic efficacy of combination therapy is less dependent on P-gp status, implying that other mechanisms, such as direct drug actions on the parasite, host immune regulation, or non-P-gp-mediated drug resistance pathways, play critical roles in determining infection outcomes.

In Fig. 1ii C,D, all drug-treated infected murine models exhibited a progressive decline in body weight (g) over a 7-day period in both WT mice (from 26.8–31.2 g to 22.8–26.6 g) and KO mice (from 23.1–26.9 g to 19.2–23.8 g). WT-resistant cohorts (from 29.4–31.2 g to 25.7–26.6 g) maintained greater body mass compared with their drug-sensitive counterparts (from 26.8–27.2 g to 22.8–24.5 g) (Fig. 1iiC).

As shown in Fig. 1iii G,I, the combination increased the hepatic and splenic indexes of WT-sensitive mice by 1.26-fold and 1.22-fold, respectively, compared with CMC-Na. In Fig. 1iii E, it also significantly elevated intestinal indices in both WT-sensitive and WT-resistant groups relative to CMC-Na (1.09-fold and 1.12-fold), with a further 1.12-fold increase in WT-resistant mice compared with monotherapy. In KO mice (Fig. 1iii H), the combination upregulated the hepatic index by 1.36-fold in sensitive mice versus the CMC-Na control. In Fig. 1iii J, it increased splenic indices in KO-sensitive and KO-resistant subgroups by 1.81-fold and 1.40-fold, respectively, compared with CMC-Na; it also increased the splenic index in KO-sensitive mice by 1.39-fold relative RIF. However, in KO-resistant mice, the combination reduced the splenic index by 1.26-fold compared with their KO-sensitive counterparts. In Fig. 1iii F, the combination increased the intestinal indices in KO-resistant mice by 1.70-fold relative to KO-sensitive controls, while decreasing them by 1.58-fold in KO-sensitive mice compared with CMC-Na. These results indicate that the combination modulates hepatic, splenic, and intestinal indices in a context-dependent manner, varying with mouse type (WT/KO), parasite sensitivity (sensitive/resistant), and the comparison group. Notably, it tends to elevate these indices in WT mice, whereas in KO mice, its effects relative to CMC-Na, monotherapy, or RIF are mixed but distinct.

### ART–CHR combination maintains potent P-gp-independent antimalarial activity against resistant *Plasmodium*, contrasting with suboptimal effects of ART alone

In WT mice (Fig. 1ivK), ART reduced parasitaemia in sensitive parasites (23.8%) but slightly increased it in resistant subgroups (25.8%), while the combination resulted in the lowest parasitaemia in resistant strains (22.5%). In KO mice (Fig. 1iv L), RIF elevated parasitaemia in both sensitive and resistant parasites (29.7% and 33.3%), whereas the combination remained effective in reducing parasitaemia (20.0%) caused by resistant parasites. Inhibition curves (Fig. 1v M,N) revealed that ART exerted greater antimalarial activity in sensitive groups with a peak inhibition rate of 34.2% on day 4, whereas only 12.0% was noted in the resistant group at the same timepoint. By day 7, the inhibition rate of ART in both the sensitive and resistant groups reached 23.3%. Notably, the combination demonstrated the highest inhibitory effects against resistant versus sensitive strains in WT and KO genotype with an inhibition rate of 33.3% and 19.8%, respectively. In contrast to the antimalarial effects of the combination, RIF showed no antimalarial activity and exacerbated infection in KO mice (Fig. 1v N).

### ART–CHR combination reshapes spatial and drug resistance–phenotypic disparities in Mrps and Bcrp mRNA expression under P-gp deficiency

To streamline data analysis, we focused on drug-resistance-related differences that can be fully reversed by ART montherapy and combination treatment, or by *Mdr1a* deficiency. The same approach was applied to subsequent analyses of the PI3K/AKT-mTOR and MAPK pathways, as well as cytokine analyses. The screening results, including fold changes, were recorded in the respective tables.

As presented in Table 2 and Supplementary Material Fig. 3, compared with their corresponding WT-sensitive subgroups, ART alone significantly reduced Mrp1 mRNA levels in the small intestines (5.05-fold decrease) of WT-resistant mice, whereas the combination increased these levels by 8.88-fold (Table 2 WT-S^1^ versus WT-R^2^). In blood samples, monotherapy increased Mrp1 mRNA expression by 1.93-fold, whereas the combination decreased it by 5.57-fold. Compared with ART alone, the combination treatment reduced Mrp1 mRNA expression in the intestines and spleens of the WT-sensitive groups by 21.47-fold and 1.04-fold, respectively, while increasing its mRNA levels in blood by 1.21-fold. In contrast, in the WT-resistant group, the combination increased intestinal and splenic Mrp1 mRNA expression by 2.09-fold and 1.82-fold, respectively, alongside an 8.91-fold decrease in blood levels (Table 2 WT-S^1^ versus WT-R^2^). In the WT-resistant subgroup, blood mRNA expressions of Mrp2 and Mrp4 were upregulated by ART compared with the WT-sensitive group (28.38-fold and 6.65-fold increases), whereas the combination downregulated them (6.32-fold and 1.59-fold decreases). Similarly, relative to ART alone, the combination increased blood Mrp2 (9.83-fold) and Mrp4 (1.23-fold) mRNA levels in the WT-sensitive group and decreased them (20.33-fold and 8.58-fold) in the WT-resistant group (Table 2 WT-S^1^ versus WT-R^2^). ART decreased Mrp5 mRNA expression in the blood and splenic samples of WT-resistant groups (1.82-fold and 1.49-fold decreases), whereas the combination increased it (1.35-fold and 1.91-fold increases) compared with WT-sensitive groups. Hepatic Bcrp mRNA levels were downregulated by ART (1.37-fold decrease) but upregulated by the combination (1.51-fold increase).

**Table 2:** ART–CHR combination reshapes spatial and drug resistance-phenotypic disparities in Mrps (Mrp1, 2, 4, and 5) and Bcrp mRNA expressions under P-gp deficiency (*n* = 4 mice per group)

Comparison	Biosample	Mrp1 mRNA		Mrp2 mRNA		Mrp4 mRNA	
		ART	ART–CHR	ART	ART–CHR	ART	ART–CHR
WT-S^1^ vs. WT-R^2^	Intestine^a^	22.097	1.029	–	–	–	–
	Intestine^b^	4.379	9.133	–	–	–	–
	Fold changes	5.05^***↓^	8.88^***↑^	–	–	–	–
	Blood^a^	0.324	0.391	0.006	0.059	0.195	0.240
	Blood^b^	0.624	0.070	0.183	0.009	1.295	0.151
	Fold changes	1.93^*↑^	5.57 ^***↓^	28.38^**↑^	6.32^**↓^	6.65^***↑^	1.59^***↓^
KO-S^a^ versus KO-R^b^	Liver^a^	7.811	1.037	–	–	–	–
	Liver^b^	0.205	6.863	–	–	–	–
	Fold changes	38.03^***↓^	6.62^***↑^	–	–	–	–
WT-S^a^ versus KO-S^b^	Liver^a^	3.450	7.701	–	–	2.387	6.739
	Liver^b^	7.298	0.969	–	–	6.002	0.528
	Fold changes	2.12^***↑^	7.95^***↓^	–	–	2.51^***↑^	12.77^**↓^
WT-R^a^ vs. KO-R^b^	Liver^a^	4.857	2.299	0.017	0.203	–	–
	Liver^b^	0.306	10.230	0.589	0.044	–	–
	Fold changes	15.86^***↓^	4.45^***↑^	34.64^***↑^	4.57^***↓^	–	–
	Blood^a^	1.473	0.156	–	–	–	–
	Blood^b^	0.586	0.936	–	–	–	–
	Fold changes	2.51^*↓^	5.99^*↑^	–	–	–	–
	Spleen^a^	0.268	0.489	–	–	0.424	0.889
	Spleen^b^	0.512	0.191	–	–	0.729	0.638
	Fold changes	1.91^**↑^	2.56^**↓^	–	–	1.72^**↑^	1.39^**↓^

Comparison	Biosample	Mrp5 mRNA		Bcrp mRNA	
		ART	ART–CHR	ART	ART–CHR
WT-S^a^ vs. WT-R^b^	Liver^S^	–	–	8.476	6.336
	Liver^R^	–	–	6.190	9.584
	Fold changes	–	–	1.37^*↓^	1.51^*↑^
	Blood^S^	1.321	0.396	–	–
	Blood^R^	0.725	0.534	–	–
	Fold changes	1.82^***↓^	1.35^*↑^	–	–
	Spleen^S^	0.631	0.312	–	–
	Spleen^R^	0.423	0.596	–	–
	Fold changes	1.49^*↓^	1.91^**↑^	–	–
KO-S^a^ versus KO-R^b^	Spleen^S^	0.404	1.207	–	–
	Spleen^R^	1.937	0.235	–	–
	Fold changes	4.79^***↑^	5.15^**↓^	–	–
WT-R^a^ versus KO-R^b^	Spleen^S^	9.883	13.929	–	–
	Spleen^R^	17.911	2.170	–	–
	Fold changes	1.81^***↑^	6.42^***↓^	–	–

*WT-S* WT-sensitive group, *WT-R* WT-resistant group, *KO-S* KO-sensitive group, *KO-R* KO-resistant group

^*^P < 0.05, ^**^P < 0.01, and ^***^P < 0.001

“–” indicates consistent changes or no significant difference between ART alone and combination. An upward arrow “↑” indicates upregulation, and a downward arrow “↓” indicates downregulation

The superscript letters “a” and “b” in the group comparison correspond to the sources of biological samples (the same as below)

The ablation of P-gp eliminated the differences in intestinal, blood, and splenic Mrp1 mRNA expression between WT-sensitive and WT-resistant groups, whether under ART alone or the combination therapy (Table 2 KO-S^1^ versus KO-R^2^). Notably, in contrast with KO-sensitive subgroups, ART decreased hepatic Mrp1 mRNA levels by 38.03-fold in KO-resistant groups, whereas the combination increased them by 6.62-fold. Meanwhile, relative to ART alone, the combination treatment reduced these levels in the livers by 7.60-fold in KO-sensitive groups and increased them by 33.48-fold in KO-resistant groups. Additionally, ART increased Mrp5 mRNA levels in the spleens of KO-resistant groups by 4.79-fold and the combination decreased them by 5.15-fold. Compared with monotherapy, the combination increased its mRNA levels by 2.99-fold but decreased them by 8.24-fold (Table 2 KO-S^1^ versus KO-R^2^). RIF decreased hepatic and blood Mrp1 mRNA levels in the KO-resistant groups by 21.17- and 1.24-fold and increased those in the intestines and spleens by 2.50- and 3.57-fold (Supplementary Material Fig. 3 ii).

The mRNA levels of Mrp1 and Mrp4 in the livers of KO-sensitive groups were upregulated by ART (2.12-fold and 2.51-fold increase) but downregulated by the combination treatment (7.95-fold and 12.77-fold decreases), compared with those in WT-sensitive mice (Table 2 WT-S^1^ versus KO-S^2^). Compared with monotherapy, the combination increased them in the WT-sensitive groups by 2.23-fold and 2.82-fold, whereas decreased those in the KO-resistant groups by 7.53-fold and 11.37-fold, respectively (Table 2 WT-S^1^ versus KO-S^2^).

Notably, compared with WT-resistant groups, monotherapy and combination elicited diametrically opposed trends in Mrp1 mRNA levels in KO-resistant groups across hepatic, blood, and splenic specimens: ART alone decreased hepatic and blood levels by 15.86-fold and 2.51-fold (versus 4.45-fold and 5.99-fold increases with combination), and increased splenic levels by 1.91-fold (versus a 2.56-fold decrease with combination) (Table 2 WT-R^1^ versus KO-R^2^). Hepatic Mrp2 expressions in the KO-resistant groups were increased by 34.64-fold with ART alone and decreased by 4.57-fold with the combination treatment, mirroring the regulatory patterns observed in splenic Mrp4 (1.72-fold increase and 1.39-fold decrease) and Mrp5 (1.81-fold increase and 6.42-fold decrease) compared with KO-sensitive groups (Table 2 WT-R^1^ versus KO-R^2^).

Overall, monotherapy and combination exhibit distinct, often opposing regulatory effects on the mRNA levels of Mrp1 as well as Mrp4 and Mrp5 across different tissues in both WT and KO mice. These differential effects are influenced by mouse genotype (WT versus KO), sensitivity/resistance status, and tissue type, which further highlights the context-dependent differences between ART alone and combination treatment.

### Opposite effects of monotherapy and combination on ROS/GSH balance in which P-gp deficiency and parasite resistance involve

**Fig 2.**
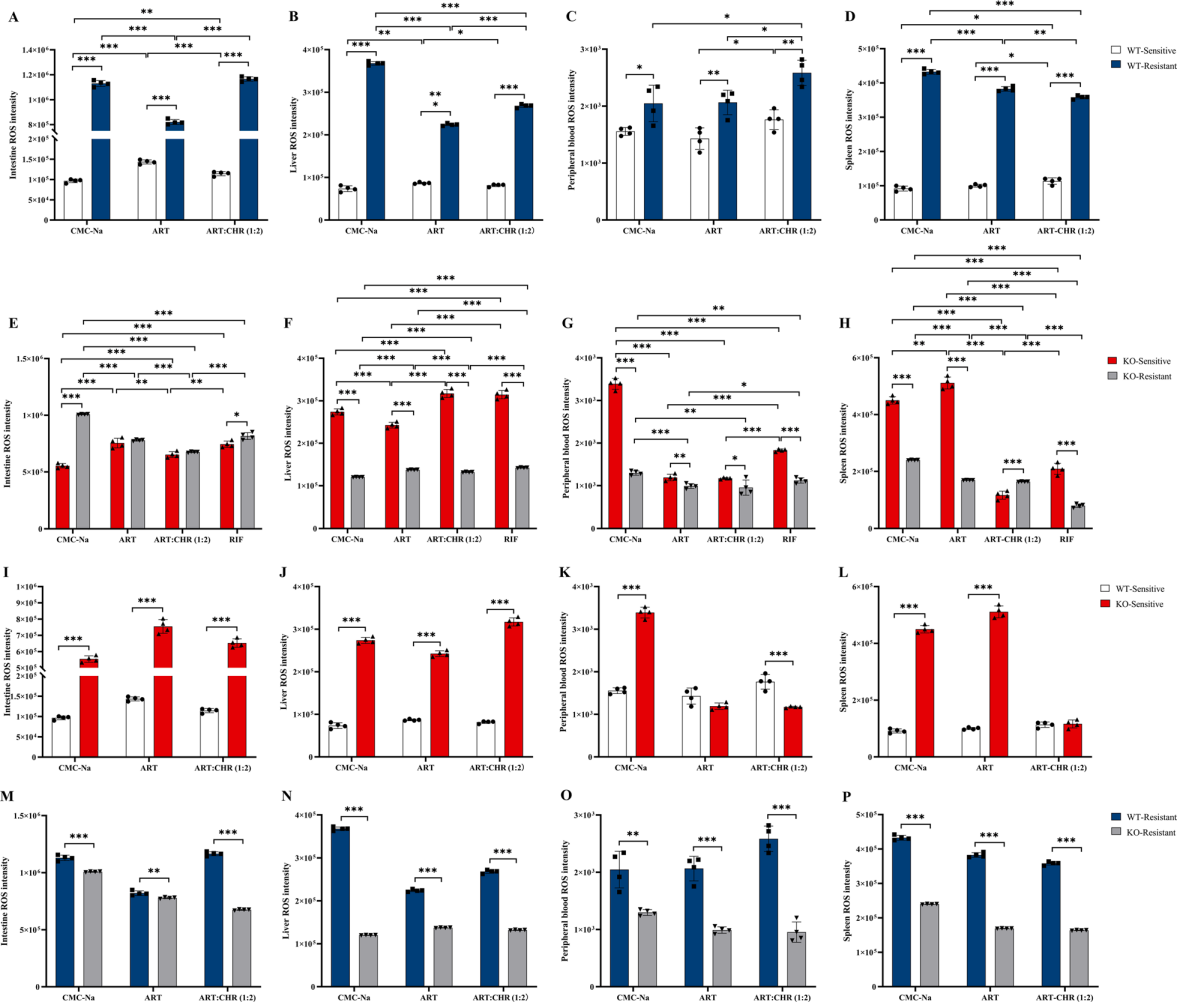
Opposite effects of monotherapy and combination on ROS levels in which P-gp deficiency and parasite resistance involve (*n* = 4 mice per group). All data were presented as $\overline{x}$ ± standard deviation (SD). The data were subjected to one-way analysis of variance (ANOVA) to detect significant differences among study groups. The Student–Newman–Keuls (SNK) was applied to determine difference between means with significance levels set at* P* < 0.05, *P* < 0.01, and *P* < 0.001. **A**, **E**, **I**, **M** Intestine samples; **B**, **F**, **J**, **N** liver samples; **C**, **G**, **K**, **O** blood samples; **D**, **H**, **L**, **P** spleen samples. CMC-Na: carboxymethyl cellulose sodium, 13 mL/kg; ART: monotherapy, 40 mg/kg; ART–CHR (1:2): artemisinin–chysosplenetin combination in ratio of 1:2, 40:80 mg/kg; RIF: rifampicin, 210 mg/kg. In the bar chart, WT-sensitive versus WT-resistant groups are distinguished by white and blue; KO-sensitive versus KO-resistant by red and gray; WT-sensitive versus KO-sensitive by white and red; and WT-resistant versus KO-resistant by blue and gray, ensuring clear differentiation. The same as below

Notably, across all drug treatment modalities, ROS levels in both the blood and tissues of WT-resistant groups were consistently elevated compared with those of WT-sensitive counterparts, with the fold changes ranging from 1.17 to 10.18 (Fig. 2A–D). Genetic ablation of the *Abcb1a* gene caused near-complete phenotypic reversal, namely a significant down-regulated ROS accumulation, with the fold changes ranging from 1.20 to 3.02, in liver, blood, and spleen of resistant subgroups versus the sensitive groups, independent of therapeutic modalities (Fig. 2E–H). Notably among these, the combination still enhanced splenic ROS intensity in KO-resistant groups (Fig. 2H), representing exceptional cases. When comparing KO-sensitive with WT-sensitive groups, ROS levels in the tissues were consistently increased by CMC-Na (5.73-fold increase in the intestine, 3.72-fold increase in the liver, and 4.89-fold in the spleen), ART alone (3.72-fold in the intestine, 2.79-fold in the liver, and 3.89-fold in the spleen), and combination (4.89-fold in the intestine, 5.11-fold in the liver, and 1.02-fold in the spleen) (Fig. 2I–L). Critically, KO-resistant subgroups demonstrated uniform suppression of ROS generation across all treatments with the fold of changes ranging from 1.05 to 3.06, in stark contrast to WT-resistant counterparts (Fig. 2M–P).

**Fig 3.**
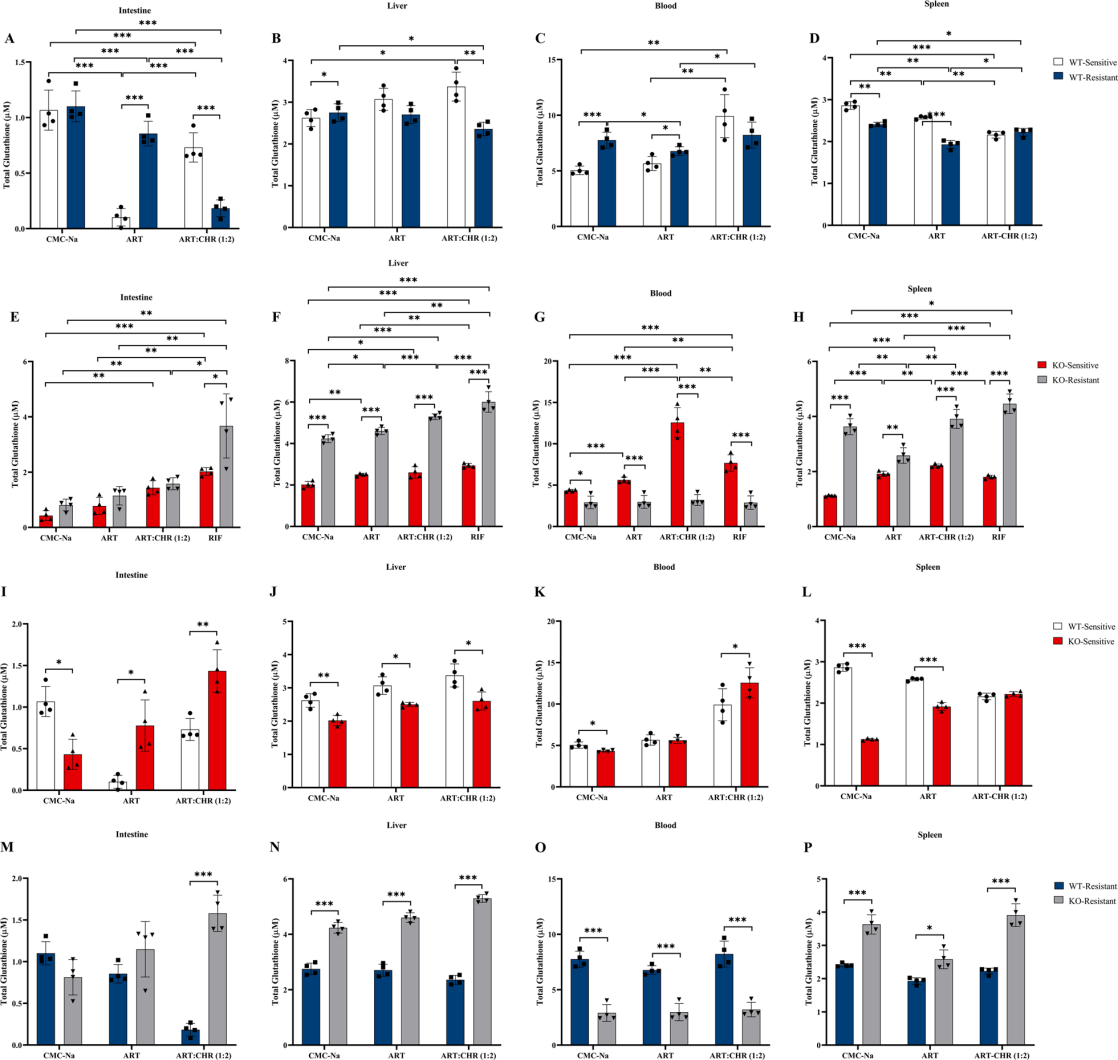
Opposite effects of monotherapy and combination on GSH levels in which P-gp deficiency and parasite resistance involve (*n* = 4 mice per group). All data were presented as $\overline{x}$ ± standard deviation (SD). The data were subjected to one-way analysis of variance (ANOVA) to detect significant differences among study groups. The Student–Newman–Keuls (SNK) was applied to determine difference between means, with significance levels set at* P* < 0.05, *P* < 0.01, and *P* < 0.001. **A**, **E**, **I**, **M** Intestine samples; **B**, **F**, **J**, **N** liver samples; **C**, **G**, **K**, **O** blood samples; **D**, **H**, **L**, **P** spleen samples

GSH levels exhibited significant spatial variations in blood and tissues between WT-sensitive and WT-resistant groups under monotherapy or combination therapy, as illustrated in Fig. 3A–D. As shown in Fig. 3A, compared with the sensitive group, ART treatment significantly elevated total GSH levels by 8.37-fold in the small intestine of the resistant group, whereas the combination therapy group exhibited a marked reduction (3.98-fold). This suggests that ART, with or without CHR, exerted diametrically opposing regulatory effects on GSH biosynthesis in the intestines of *P. berghei*-infected mice, depending on parasite’s sensitivity/resistance status. Similar trends were observed in blood samples (Fig. 3C), although the magnitude of significant differences was less pronounced (1.20-fold and 1.21-fold, respectively). Compared with KO-sensitive groups, KO-resistant groups showed significantly elevated GSH levels in the liver (2.10-fold, 1.84-fold, 2.03-fold, and 2.04-fold) and spleen (3.23-fold, 1.35-fold, 1.76-fold, and 2.48-fold) across all drug treatments (CMC-Na, ART alone, combination, and RIF), whereas blood GSH levels were consistently decreased (1.49-fold, 1.89-fold, 3.92-fold, and 2.66-fold) (Fig. 3E–H). Similar to the alterations in the ROS system, P-gp deficiency exerted opposite regulatory effects on GSH level differences in the blood and tissues of mice infected with sensitive and resistant *Plasmodium* parasites, with consistent effects for ART alone and combination therapy (Fig. 3I–P). Specifically, in KO-sensitive groups, hepatic GSH levels were downregulated by ART alone and its combination by 1.23-fold and 1.25-fold, respectively, compared with their WT-sensitive counterparts (Fig. 3J,L), whereas intestinal GSH levels were significantly upregulated by 7.61-fold and 1.96-fold, respectively (Fig. 3I,J). In contrast, KO-resistant groups exhibited upregulated hepatic and splenic GSH levels across all drug treatments, with fold changes ranging from 1.34 to 2.24 relative to WT-resistant controls (Fig. 3N,P), coupled with downregulated blood GSH levels (2.28-fold to 2.67-fold) (Fig. 3O).

Hence, monotherapy and combination show similar effects on ROS and GSH levels in most cases, with the exception that the combination reversed ART-induced ROS accumulation in the intestine and blood of WT-resistant mice (versus WT-sensitive mice) and splenic GSH reduction in resistant KO mice (versus KO-sensitive mice). P-gp deficiency profoundly alters these patterns, suppressing ROS in resistant KO mice and reversing GSH level differences between sensitive and resistant groups compared with WT, with tissue-specific variations. These findings highlight that P-gp dictates redox (ROS/GSH) regulation in *Plasmodium* infection, with the combination’s effects tied to P-gp-dependent mechanisms.

### ART–CHR combination inhibits PI3K/AKT-mTOR signaling pathway dysregulated by *Mdr1a* deficiency

In Table 3 (WT-S^1^ versus WT-R^2^) and Supplementary Material Fig. 4, compared with the WT-sensitive controls, monotherapy decreased AKT and mTOR mRNA levels in the intestines of the WT-resistant groups by 3.47-fold and 3.82-fold, respectively, whereas the combination increased them by 1.59-fold and 2.49-fold. In contrast, ART increased blood AKT mRNA levels by 8.34-fold, whereas the combination decreased them by 1.71-fold. When compared with monotherapy, combination therapy decreased intestinal AKT and mTOR mRNA and blood AKT mRNA in the WT-sensitive groups by 2.63-, 4.19-, and 4.42-fold, alongside 2.09-, 2.27-, and 3.23-fold increases in the WT-resistant groups (Table 3 WT-S^1^ versus WT-R^2^). P-gp deficiency induced opposite change trends of hepatic and blood PI3K mRNA levels in KO-resistant groups compared with KO-sensitive controls between ART and combination as presented in Table 3 (KO-S^1^ versus KO-R^2^): ART and RIF decreased hepatic PI3K mRNA levels by 84.00-fold and 21.99-fold but increased those in blood by 2.19-fold and 14.45-fold (versus 1.89-fold increase and 12.81-fold decrease by the combination). ART and RIF monotherapy decreased mTOR mRNA levels in the intestines by 1.68-fold and 1.84-fold while increased those in the blood by 3.62-fold and 2.23-fold (versus 2.32-fold increase and 1.52-fold decrease by the combination) (Table 3 KO-S^1^ versus KO-R^2^). In Table 3 (WT-S^1^ versus KO-S^2^), compared with the WT-sensitive controls, monotherapy increased hepatic and splenic PI3K mRNA levels in the KO-sensitive by 2.27-fold and 6.70-fold, whereas the combination decrease them by 6.36-fold and 2.41-fold, respectively. Its levels in the intestines were also increased by monotherapy (3.04-fold increase) and decreased by the combination (6.60-fold decrease) when compared KO-resistant groups with the WT-resistant controls (Table 3 WT-R^1^ versus KO-R^2^).

**Table 3:** P-gp-independent spatially antagonistic regulation of ART alone and combination therapy on PI3K/AKT-mTOR pathway (*n* = 4 mice per group)

Comparison	Biosample	PI3K mRNA		AKT mRNA		mTOR mRNA	
		ART	ART–CHR	ART	ART–CHR	ART	ART–CHR
WT-S^1^ versus WT-R^2^	Intestine^1^	–	–	1.087	0.413	4.061	0.969
	Intestine^2^	–	–	0.314	0.656	1.064	2.416
	Fold changes	–	–	3.47^**↓^	1.59^*↑^	3.82^***↓^	2.49^***↑^
	Liver^1^	–	–	–	–	–	–
	Liver^2^	–	–	–	–	–	–
	Fold changes	–	–	–	–	–	–
	Blood^1^	–	–	0.293	0.945	–	–
	Blood^2^	–	–	2.442	0.553	–	–
	Fold changes	–	–	8.34^***↑^	1.71^***↓^	–	–
	Spleen^1^	–	–	–	–	–	–
	Spleen^2^	–	–	–	–	–	–
	Fold changes	–	–	–	–	–	–
KO-S^1^ vs. KO-R^2^	Intestine^1^	–	–	–	–	1.538	1.941
	Intestine^2^	–	–	–	–	0.916	4.503
	Fold changes	–	–	–	–	1.68^**↓^	2.32^*↑^
	Liver^1^	8.631	0.402	–	–	–	–
	Liver^2^	0.103	0.761	–	–	–	–
	Fold changes	84.00^***↓^	1.89^***↑^	–	–	–	–
	Blood^1^	2.421	7.944	–	–	0.549	1.315
	Blood^2^	5.310	0.620	–	–	1.986	0.864
	Fold changes	2.19^**↑^	12.81^***↓^	–	–	3.62^***↑^	1.52^**↓^
	Spleen^1^	–	–	–	–	–	–
	Spleen^2^	–	–	–	–	–	–
	Fold changes	–	–	–	–	–	–
WT-S^1^ versus KO-S^2^	Liver^1^	0.720	0.484	–	–	–	–
	Liver^2^	1.633	0.076	–	–	–	–
	Fold changes	2.27^***↑^	6.36^***↓^	–	–	–	–
	Blood^1^	–	–	–	–	–	–
	Blood^2^	–	–	–	–	–	–
	Fold changes	–	–	–	–	–	–
	Spleen^1^	0.154	0.065	–	–	–	–
	Spleen^2^	1.031	0.027	–	–	–	–
	Fold changes	6.70^***↑^	2.41^**↓^	–	–	–	–
WT-R^1^ versus KO-R^2^	Intestine^1^	0.400	0.766	–	–	–	–
	Intestine^2^	1.215	0.116	–	–	–	–
	Fold changes	3.04^**↑^	6.60^***↓^	–	–	–	–

*WT-S* WT-Sensitive group, *WT-R* WT-Resistant group, *KO-S* KO-Sensitive group, *KO-R* KO-Resistant group

The superscript numbers “1” and “2” in the group comparison correspond to the sources of biological samples

“–” indicates consistent changes or no significant difference between ART alone and combination. An upward arrow “↑” indicates upregulation, and a downward arrow “↓” indicates downregulation

*P < 0.05, **P < 0.01, and ***P < 0.001

**Fig. 4:**
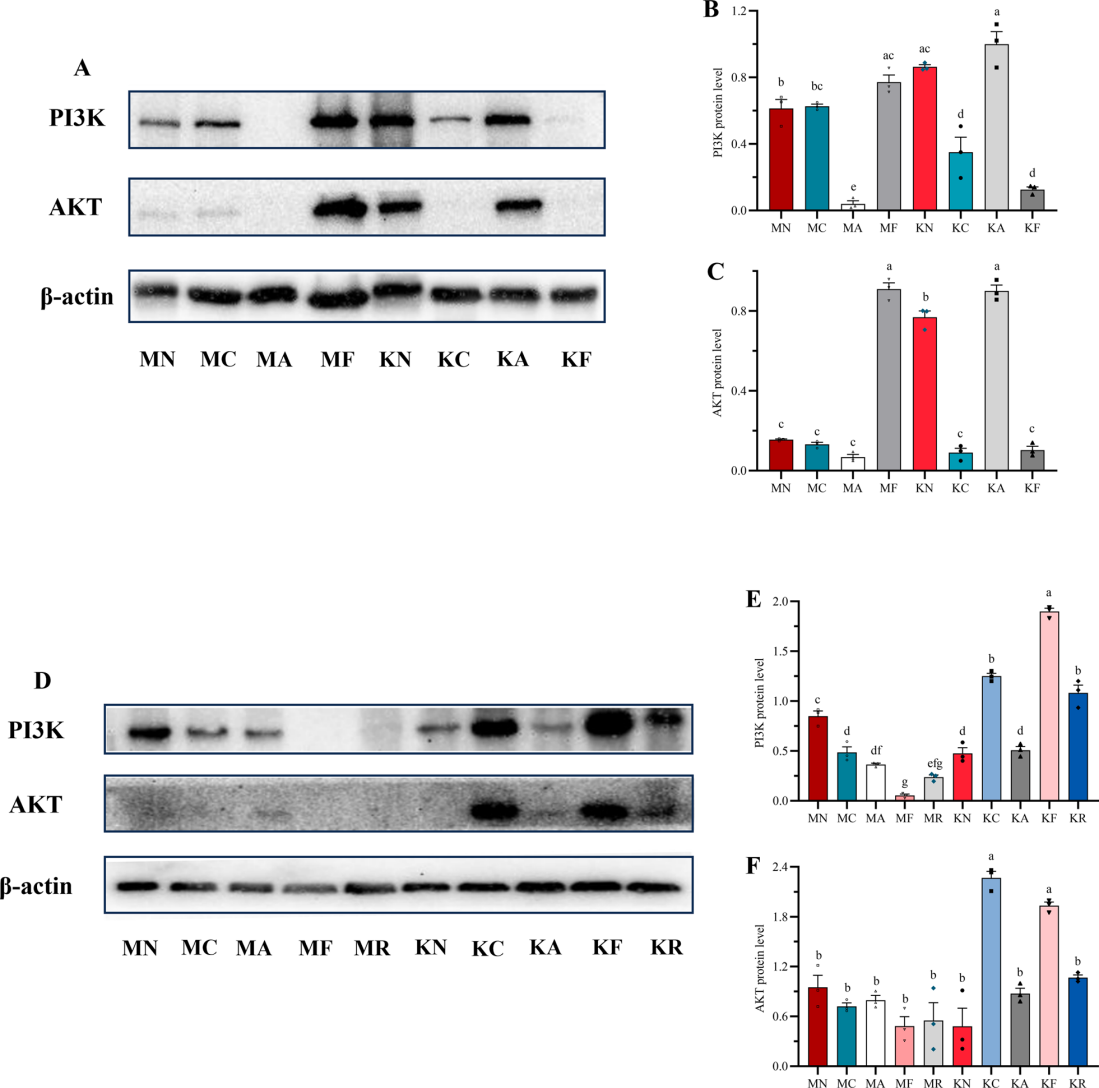
ART–CHR combination inhibits PI3K/AKT-mTOR protein expression dysregulated by *Mdr1a* deficiency (*n* = 3 mice per group). All data were presented as $\overline{x}$ ± standard deviation (SD). The data were subjected to one-way analysis of variance (ANOVA) to detect significant differences among study groups. The Student–Newman–Keuls (SNK) was applied to determine difference between means, with significance levels set at* P* < 0.05. Different lowercase letters are used to intuitively distinguish significant differences between data groups, whereas the same letters indicate no significant difference between groups. **A**, **B**, **C** Original WB bands and relative protein expression levels of PI3K and AKT in the small intestine of each WT group; **D**, **E**, **F** original WB bands and relative protein expression levels of PI3K and AKT in the small intestine of each KO group. MN: sensitive groups without drug treatment (negative control); MC: sensitive groups under CMC-Na treatment (negative control); MA: sensitive groups under ART treatment; MF: sensitive groups under the combination treatment; KN: resistant groups without drug treatment; KC: resistant groups under CMC-Na treatment; KA: resistant groups under ART treatment; KF: resistant groups under the combination treatment; MR: KO-Sensitive groups under RIF treatment; KR: KO-Resistant groups under RIF treatment

In the small intestinal milieu, the untreated WT-resistant group (naïve to blank solvent vehicle) displayed significantly elevated PI3K/AKT protein expression by 2.04-fold and 3.74-fold, respectively, compared with the untreated WT-sensitive counterpart (Fig. 4A–C). Notably, in WT-resistant mice, CMC-Na (the blank solvent vehicle) suppressed PI3K and AKT protein levels by 1.79-fold and 1.20-fold, relative to the corresponding WT-sensitive controls. Monotherapy upregulated PI3K/AKT protein expressions in WT-resistant mice by 16.00-fold and 10.63-fold, respectively, compared with the WT-sensitive counterparts (Fig. 4A–C). In contrast, the combination therapy decreased these proteins in WT-resistant mice by 6.18-fold and 7.23-fold, respectively (Fig. 4A–C). These findings suggested a positive correlation between ART resistance and PI3K/AKT hyperactivation, which is involved in reversal of ART resistance by CHR, while antagonizing ART-induced suppression in sensitive *Plasmodium*. Genetic ablation of *Mdr1a* completely inverted intestinal PI3K/AKT expression profiles in resistant groups across all experimental settings, with 1.87- and 2.97-fold decreases in untreated groups, 2.71- and 3.38-fold increases in CMC-Na groups, and 63.00- and 4.02-fold increases in combination groups, respectively, compared with their corresponding sensitive groups (Fig. 4D–F). There was no significant difference in either PI3K or AKT between KO-resistant and KO-sensitive groups following ART treatment (Fig. 4D–F). RIF increased PI3K protein expression in KO-resistant groups by 4.92-fold relative to KO-sensitive groups (Fig. 4D–F). Collectively, monotherapy and combination oppositely regulated PI3K/AKT-mTOR pathway in a tissue-, genotype-, and sensitivity/resistance-dependent manner, with CHR reversing ART-induced changes through PI3K/AKT inhibition that is contingent on P-gp functionality.

### P-gp-independent spatially antagonistic regulation of ART alone and combination therapy on MAPK pathway

In both WT-sensitive and WT-resistant mouse subgroups, monotherapy and combination therapy exerted completely opposite regulatory effects on blood ERK, JNK, and p38 gene levels (Table 4 and Supplementary Material Fig. 5). Specifically, compared with WT-sensitive controls, ART up-regulated blood ERK, JNK, and p38 mRNA in WT-resistant mice by 4.90-, 8.95-, and 8.15-fold, respectively, whereas the combination therapy down-regulated these expressions by 2.62-, 2.58-, and 2.31-fold compared with the sensitive counterparts (Table 4 WT-S^1^ versus WT-R^2^). This inverse regulation was similarly observed in blood samples from KO-sensitive and KO-resistant mice (Table 4 KO-S^1^ vs. KO-R^2^), with fold changes ranging from 1.91- to 16.69-fold for all three genes. RIF therapy led to consistent change trends with those treated with ART. Intestinal ERK mRNA expressions were increased by monotherapy (1.47-fold increase) and those in the livers were decreased (2.00-fold decrease), whereas the combination decreased those in the intestines by 1.39-fold and increased those in the livers by 2.11-fold, when comparing WT- and KO-sensitive mice (Table 4 WT-S^1^ versus KO-S^2^). In KO mice infected with resistant parasites versus their corresponding WT groups, ART still increased intestinal ERK (1.63-fold) and p38 mRNA (2.14-fold) expressions, whereas the combination therapy decreased them by 1.58- and 1.90-fold, respectively (Table 4 WT-R^1^ versus KO-R^2^).

**Table 4:** P-gp-independent spatially antagonistic regulation of ART alone and combination therapy on MAPK pathway (*n* = 4 mice per group)

Comparison	Biosample	ERK mRNA		JNK mRNA		p38 mRNA	
		ART	ART–CHR	ART	ART–CHR	ART	ART–CHR
WT-S^1^ versus WT-R^2^	Blood^1^	0.552	2.047	0.329	1.818	0.277	1.612
	Blood^2^	2.706	0.780	2.941	0.705	2.261	0.698
	Fold changes	4.90^***↑^	2.62^***↓^	8.95^***↑^	2.58^***^↓	8.15^***^↑	2.31^***^↓
KO-S^1^ versus KO-R^2^	Blood^1^	0.566	1.838	0.282	2.114	0.438	0.651
	Blood^2^	1.337	0.246	0.585	0.127	0.837	0.070
	Fold changes	2.36^***^↑	7.47^***^↓	2.07^***^↑	16.69^***^↓	1.91^***^↑	9.34^***^↓
WT-S^1^ vs. KO-S^2^	Intestine^1^	0.767	0.865	–	–	–	–
	Intestine^2^	1.121	0.623	–	–	–	–
	Fold changes	1.47^***^↑	1.39^**^↓	–	–	–	–
	Liver^1^	1.278	0.466	–	–	–	–
	Liver^2^	0.638	0.987	–	–	–	–
	Fold changes	2.00^***↓^	2.11^***^↑	–	–	–	–
WT-R^1^ versus KO-R^2^	Intestine^1^	0.383	0.410	–	–	0.501	0.797
	Intestine^2^	0.623	0.259	–	–	1.074	0.421
	Fold changes	1.63^***↑^	1.58^*↓^	–	–	2.14^***^↑	1.90^***^↓

*WT-S* WT-Sensitive group, *WT-R* WT-Resistant group, *KO-S* KO-Sensitive group, *KO-R* KO-Resistant group

The superscript numbers “1” and “2” in the group comparison correspond to the sources of biological samples

“–” indicates consistent changes or no significant difference between ART alone and combination. An upward arrow “↑” indicates upregulation, and a downward arrow “↓” indicates downregulation

*P < 0.05, **P < 0.01, and ***P < 0.001

Hence, there were no significant differences in the regulatory trends of ERK, JNK, and p38 gene expressions between monotherapy and combination therapy regardless of the presence or absence of *Mdr1a*. These findings suggest that the tissue and parasite genotype-specific differential regulation of the MAPK pathway by CHR in combination therapy may be independent of P-gp.

### P-gp modulates hemoglobin level responses to monotherapy and combination in WT and KO mice with sensitive and resistant parasite strains

**Fig 5.**
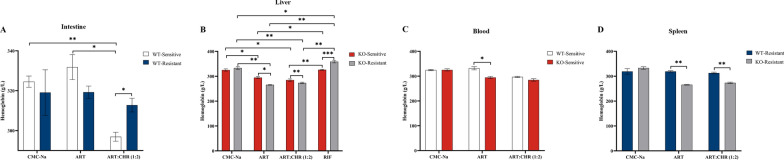
P-gp modulates hemoglobin level responses to monotherapy and combination in WT and KO mice with sensitive and resistant parasite strains (*n* = 4 mice per group). All data were presented as $\overline{x}$ ± standard deviation (SD). The data were subjected to one-way analysis of variance (ANOVA) to detect significant differences among study groups. The Student–Newman–Keuls (SNK) was applied to determine difference between means with significance levels set at* P* < 0.05, *P* < 0.01, and *P* < 0.001. **A** Intestine samples;** B** liver samples; **C** blood samples; **D** spleen samples

Compared with monotherapy, the combination therapy elicited a significant 1.12-fold decrease in hemoglobin concentration in WT-sensitive subgroups (296.92 g/L versus 331.86 g/L) (Fig. 5A). Concurrently, this combinatorial regimen markedly elevated hemoglobin levels by 1.05-fold in WT-resistant mice (312.78 g/L) (Fig. 5A), an effect partially mitigated by genetic ablation of P-gp (resulting in a 1.04-fold decrease). RIF treatment significantly enhanced hemoglobin levels in both sensitive (326.15 g/L) and resistant (358.88 g/L) cohorts, relative to the monotherapy (294.69 and 265.59 g/L) and combination therapy groups (284.69 and 273.48 g/L), respectively (Fig. 5B). In KO-sensitive mice, ART administration induced an 1.13-fold reduction in hemoglobin levels compared with WT-sensitive counterparts; similarly, both monotherapy and combination therapy resulted in 1.20- and 1.14-fold decreases in hemoglobin levels in KO-resistant phenotypes, respectively (Fig. 5C,D). Collectively, these findings implicate a potential role of P-gp in modulating hemoglobin level responses to drugs.

### Opposing regulation of cytokine expression by monotherapy and combination in a spatial-, parasite phenotype-, and P-gp-dependent manner

In the WT-sensitive and WT-resistant groups, monotherapy and combination treatment exerted diametrically opposed regulatory effects on splenic TNF-*α*, IFN-*γ*, and IL-1*β* mRNA expression profiles (Table 5 and Supplementary Material Fig. 6). Specifically, compared with WT-sensitive controls, ART upregulated these three cytokines in the spleens of WT-resistant mice (24.05-, 6.04-, and 1.14-fold increases), whereas combination treatment decreased them by 1.97-, 3.12-, and 1.22-fold, respectively (Table 5 WT-S^1^ versus WT-R^2^).

**Table 5:** Opposing regulation of cytokine expressions by monotherapy and combination in a spatial-, parasite phenotype-, and P-gp-dependent manner (*n* = 4 mice per group)

Comparison	Biosample	TNF-*α* mRNA		IL-1*β* mRNA		IFN-*γ* mRNA	
		ART	ART–CHR	ART	ART–CHR	ART	ART–CHR
WT-S^1^ versus WT-R^2^	Spleen^1^	0.334	1.318	0.400	1.091	0.880	0.219
	Spleen^2^	8.019	0.669	2.414	0.350	1.002	0.180
	Fold changes	24.05^**↑^	1.97^**↓^	6.04^**↑^	3.12^**↓^	1.14^*↑^	1.22^*↓^
KO-S^1^ versus KO-R^2^	Intestine^1^	–	–	–	–	0.698	2.539
	Intestine^2^	–	–	–	–	2.564	0.233
	Fold changes	–	–	–	–	3.67^***↑^	10.90^***↓^
	Blood^1^	0.347	0.361	3.523	8.265	0.220	2.047
	Blood^2^	0.899	0.103	5.444	0.219	5.093	0.193
	Fold changes	2.59^***↑^	3.52^***↓^	1.55^*↑^	37.79^***↓^	23.11^***↑^	10.60^***^↓
WT-S^1^ versus KO-S^2^	Liver^1^	0.394	1.669	–	–	0.489	1.572
	Liver^2^	3.425	0.488	–	–	1.217	0.142
	Fold changes	8.70^***↑^	3.42^**↓^	–	–	2.49^*↑^	11.21^***↓^
WT-R^1^ vs. KO-R^2^	Intestine^1^	–	–	–	–	0.834	1.507
	Intestine^2^	–	–	–	–	2.513	0.228
	Fold changes	–	–	–	–	3.02^***^↑	6.57^**^↓
	Blood^1^	–	–	–	–	0.510	0.953
	Blood^2^	–	–	–	–	3.451	0.108
	Fold changes	–	–	–	–	6.77^***^↑	8.82^***^↓

*WT-S* WT-Sensitive group, *WT-R* WT-Resistant group, *KO-S* KO-Sensitive group, *KO-R* KO-Resistant group

The superscript numbers “1” and “2” in the group comparison correspond to the sources of biological samples

“–” indicates consistent changes or no significant difference between ART alone and combination. An upward arrow “↑” indicates upregulation, and a downward arrow “↓” indicates downregulation

*P < 0.05, **P < 0.01, and ***P < 0.001

*Mdr1a* ablation almost abrogated splenic cytokine inversion between KO-sensitive and KO-resistant groups induced by ART alone and combination (Table 5 KO-S^1^ versus KO-R^2^). Instead, compared with the KO-sensitive controls, ART increased TNF-*α*, IFN-*γ*, and IL-1*β* mRNA expressions in blood samples of KO-resistant mice by 2.59-, 1.55-, and 23.11-fold, whereas the combination decreased them by 3.52-, 37.79-, and 10.60-fold, respectively (Table 5 KO-S^1^ versus KO-R^2^). ART increased intestinal IFN-*γ* mRNA levels in the KO-resistant groups by 3.67-fold but combination decreased them by 10.90-fold, compared with the KO-sensitive controls (Table 5 KO-S^1^ versus KO-R^2^). Accordingly, RIF treatment increased them by 26.56-, 26.78-, and 23.53-fold, respectively (Supplementary Material Fig. 6).

In KO-sensitive mice, compared with WT-sensitive mice, ART increased hepatic TNF-*α* and IFN-*γ* by 8.70- and 2.49-fold, whereas the combination therapy decreased them by 3.42- and 11.21-fold, respectively (Table 5 WT-S^1^ *vs*. KO-S^2^). Compared with WT-resistant mice, ART increased IFN-*γ* mRNA levels in the intestine and blood of KO-resistant mice by 3.02- and 6.77-fold, whereas the combination decreased them by 6.57- and 8.82-fold, respectively (Table 5 WT-R^1^ versus KO-R^2^).

In terms of results, therefore, monotherapy and combination exert opposing regulatory effects on cytokine expression in a tissue-, parasite sensitivity/resistance-, and P-gp-dependent manner, with RIF mimicking monotherapy effects in KO mice.

## Discussion

As is well known, ART resistance develops through multiple mechanisms. Among these, excessive drug efflux mediated by ABC transporters (linked to multidrug resistance, MDR) [14] and the lethal effects of heme-triggered ROS accumulation [7] are likely the two main aspects. In fact, the function of ABC transporters is not restricted to chemical efflux; they are also involved in various cancer hallmarks, including enhanced cell proliferation [26, 27], resistance to cell death [28, 29], and cell migration and invasion [30, 31]. Intriguingly, the expression of ABC transporters is indeed induced by ROS as a mechanism to counter oxidative stress, and some of these transporters are also implicated in intracellular free heme release and ROS production [32, 33]. From our perspective, the crosstalk between ABC transporters and the heme-ROS/GSH axis contributes to the formation of a more intricate loop that encompasses the dual mechanisms of ART resistance. These bidirectional interactions together form a self-reinforcing loop: ABC transporters reduce intracellular ART levels through efflux while perturbing redox balance, which in turn upregulates transporter expression and activity via ROS/GSH-dependent signaling. Concurrently, the heme-ROS/GSH axis, by regulating oxidative stress and GSH availability, not only protects cells from ART-induced oxidative damage, but also fuels the transporter-mediated efflux machinery. This integration of drug extrusion and redox adaptation generates a more robust resistance phenotype that is harder to reverse, as targeting either mechanism alone may be compensated for by the other arm of the loop. In essence, the crosstalk between ABC transporters and the heme-ROS/GSH axis transforms two independent resistance pathways into a coordinated system, thereby amplifying the ability of cells to evade ART-mediated killing.

Our previous work revealed that reversal of ART resistance by CHR may be associated with the downregulation of P-gp at both the gene and protein levels [24]. However, a critical puzzle remains unresolved. Although there is ongoing debate over whether ART acts as a P-gp substrate, most evidence suggests that it is likely a weak or atypical substrate. This raises the question of whether P-gp exerts its effects primarily as an efflux pump or through other regulatory roles in CHR’s reversal mechanism, and this issue requires further clarification. In fact, beyond its direct drug efflux function, the P-gp homologous transporter may also be responsible for heme trafficking across the plasma membrane or FV membrane in malarial parasites [34]. For instance, a *Plasmodium falciparum* ATP-binding cassette transporter, ABCC2, has been shown to be essential for the entry of liver-stage parasites into schizogony [34], which highlights the diverse roles of ABC transporters beyond drug efflux. To address this, we further compared the differences in antimalarial activity and the expression profiles of other major ABC transporters (Mrps and Bcrp) between monotherapy and combination therapy, in the blood and tissues of *Mdr1a* WT and KO mice infected with either sensitive or resistant malarial parasites.

Firstly, after *Mdr1a* KO, Mrp1 emerged as the most prominent transporter involved in the mechanism by which CHR reverses ART resistance. As a GSH transport pump, Mrp1 is widely expressed in human tissues, with predominant distribution in the lung, liver, kidney, testis, placenta, and peripheral blood cells [35]. Its transport mechanism is closely linked to GSH. In many cases, Mrp1 requires GSH to facilitate drug efflux [36]. Studies have demonstrated that Mrp1 can regulate the proinflammatory molecule leukotriene C4 to exert antioxidant defense [37], and it also protects cardiomyocytes and cardiac fibroblasts from doxorubicin-induced toxicity by modulating extracellular redox status [38].

Mrp2 is a key driver of bile flow, promoting bile secretion and increasing the lipid solubility of bile salts. Down-regulation of Mrp2 expression leads to excessive release of inflammatory cytokines, which in turn causes liver injury. Inflammatory cytokines such as IL-1 can also inhibit extracellular regulated kinase and downregulate Mrp expression, thereby forming a vicious cycle. Mrp4 and Mrp5 share only 35% identity with Mrp1 and function as nucleotide transporters. Bcrp, encoded by the ABCG2 gene, is a half-transporter that requires homodimer or oligomer formation via disulfide bonds [39, 40]. The tissue distribution of Bcrp and P-gp overlaps extensively, suggesting that they exert similar pharmacological and toxicological protective effects. In addition to transporting hydrophobic substrates, Bcrp can also carry hydrophilic conjugated organic anions [41], whereas P-gp generally transports hydrophobic compounds. This overlap in substrate specificity may enable Bcrp and P-gp to synergistically limit drug passage through tissue barriers such as the blood–brain barrier. Bcrp regulates endogenous substances (e.g., heme and porphyrin) to maintain cellular homeostasis [42]. Enhanced heme extrusion mediated by ABCG2 reduces ROS levels and mitochondrial damage, and vice versa [43–45]. As highlighted in recent studies, the roles of ABC transporters extend beyond chemotherapeutic efflux to encompass multiple other functions [46–48].

Studies have shown that artesunate-induced DNA damage in *Plasmodium falciparum* arises from oxidative stress via the generation of free radicals and ROS [49]. ART resistance is associated with the parasite’s ability to manage oxidative stress and redox-mediated processes [50]. GSH, an important endogenous antioxidant, participates in numerous critical cellular processes, including detoxification and maintenance of redox balance. Notably, the slower depletion of GSH in ART-resistant strains under ART exposure underscores the role of the GSH system in resistance [50]. ABC transporters play a pivotal role in GSH transport, facilitating its movement across membranes to maintain optimal intracellular concentrations and ensure cellular homeostasis. This transport involves multiple ABC transporters that mediate bidirectional GSH flux, exporting GSH from the cytosol or importing it into the cell, to meet metabolic demands. For instance, certain ABC transporters recognize and bind GSH, then translocate it across the membrane via their transmembrane domains. Furthermore, GSH transport is governed by diverse mechanisms, including synergistic interactions with ABC transporters and GSH’s association with other molecules (e.g., reduced GSH involves in MRP1 transport). These mechanisms collectively regulate GSH distribution and concentration, which are critical for maintaining normal cellular physiology.

Research has demonstrated that mutations in *Plasmodium falciparum* Kelch13 (PfK13) destabilize the protein structure, thereby dysregulating the PI3K/AKT signaling pathway implicated in ART resistance and impairing the binding capacity of *P. falciparum* PI3K (PfPI3K) [51]. The PI3K/AKT-mTOR pathway is one of three major cellular signal transduction pathways that play a critical role in cancer or other diseases such as asthma [52], which can be attenuated by artesunate via inhibition of this pathway. mTOR is an important kinase downstream of PI3K/AKT, regulating tumor cell proliferation, growth, survival, and angiogenesis. The pathway generally promotes cell survival by inhibiting pro-apoptotic factors or activating anti-apoptotic factors and functions in cancer stem cell self-renewal and resistance to chemotherapy or radiotherapy. Our study revealed that monotherapy and combination treatment exerted diametrically opposite regulatory effects on the protein expression levels of PI3K and AKT in the small intestines of WT-sensitive and WT-resistant mice. Specifically, monotherapy downregulated protein expression in the sensitive group while upregulating it in the resistant group, whereas the combination treatment exerted the opposite effects. Meanwhile, PI3K and AKT exhibited inconsistent, even diametrically opposite, expression patterns at the gene and protein levels in the small intestines of WT and KO mice infected with drug-sensitive or drug-resistant parasites under different drug treatments. This finding confirms that PI3K/AKT protein expression is subject to significant post-transcriptional regulation. Unfortunately, due to low protein expression or other unknown factors, we were unable to reliably quantify protein levels in other tested samples. Consequently, our comparative analysis of these differences was restricted to the small intestine.

At the mRNA level, we observed irregular expression patterns of PI3K, AKT, and mTOR genes across biological samples. In summary, spatial differentiation of these target genes was ubiquitous in WT and KO mice infected with sensitive and resistant parasites under different drug treatments. Phenotypic variations were widespread within different subgroups. In most cases, the combination treatment exerted an inverse regulatory effect on resistant parasites, suggesting that CHR may function as a homeostasis stabilizer rather than a pure inhibitor or inducer. Furthermore, *Mdr1a* KO induced reverse changes to some extent, indicating a clear correlation between P-gp and PI3K/AKT-mTOR pathway.

It has been reported that ART induces neurite outgrowth in PC12 cells through the activation of ERK and p38 MAPK, and this effect is inhibited by the MAPK/ERK kinase inhibitor PD98059 or the p38 MAPK inhibitor SB203580 [53]. The MAPK pathway is a highly conserved intracellular signaling network that transduces extracellular stimuli—including growth factors, stress signals, and inflammatory cues—to the nucleus, thereby regulating critical cellular processes such as cell proliferation, differentiation, apoptosis, and stress responses. The relationship between P-gp and MAPK regulation is not absolute, as it depends on specific biological contexts, cell types, and signaling components involved. Instead, their interplay is nuanced, differing across MAPK subfamilies, biological systems, and physiological or pathological conditions. Our work confirmed that the MAPK pathway (e.g., ERK, JNK, or p38 signaling) is regulated by the combination independently of P-gp.

Reduced hemoglobin uptake in ART-resistant PfK13 mutant parasites has been shown to result in diminished heme-based activation of the ART endoperoxide ring and reduced cytocidal activity, which acts as one of the resistance mechanisms [54, 55]. Hemoglobin and free heme are closely related molecules involved in oxygen transport and metabolism. Hemoglobin is a large, complex protein composed of four globin subunits (typically two *α* and two *β* chains in adult hemoglobin), each covalently bound to a heme group. A heme group consists of a porphyrin ring with an iron ion (Fe^2+^) at its center and this iron ion is critical for oxygen binding. Thus, hemoglobin’s oxygen-carrying capacity directly depends on its associated heme groups. Free heme refers to heme groups that are not bound to globin proteins. It exists in a “free” state, unassociated with the globin subunits of hemoglobin. Hemoglobin’s primary role is to transport oxygen from the lungs to tissues and carry carbon dioxide back to the lungs. The globin subunits stabilize the heme groups and regulate their oxygen-binding affinity. In contrast, free heme is not involved in physiological oxygen transport. In fact, unbound heme is highly reactive: its iron ion can catalyze the production of ROS, causing oxidative damage to cells and tissues. For this reason, free heme is typically sequestered or degraded by proteins such as hemopexin (which binds free heme and facilitates its removal) to prevent toxicity. When RBCs are degraded (e.g., in the spleen), hemoglobin is broken down. The globin subunits are recycled, and the heme groups are metabolized into biliverdin (then bilirubin) by heme oxygenase. If this process is disrupted (e.g., due to hemolysis), excess heme is released into the circulation as free heme. Elevated free heme levels (e.g., in hemolytic anemia or tissue injury) are associated with inflammation and organ damage, as free heme activates immune responses and induces oxidative stress. Under normal conditions, hemoglobin’s stability prevents the release of free heme. Its structure ensures heme remains bound and functional, avoiding the toxic effects of free heme. In summary, free heme is an essential component of hemoglobin, but only when bound to globin proteins does it contribute to physiological oxygen transport. Free heme, in contrast, is a potentially harmful byproduct requiring tight regulation. As shown in Fig. 5, the combination treatment restrained hemoglobin production in peripheral blood of the sensitive groups but promoted it in the resistant groups. When *Mdr1a* was knocked out, hemoglobin synthesis might be decreased in both strains of parasite-infected mice under ART alone and its combination. Therefore, we hypothesize that the regulation of CHR on hemoglobin might be closely related to the involvement of P-gp. Hence, CHR might improve anemia induced by resistant strains of parasites but worsen it in the sensitive groups.

Immune cell chemotaxis to pathogen infection is critical, but excessive activation of the innate immune system potentially causes a damaging invasion of immune cells into tissues and a consequent surge of cytokines [56]. IFN-*γ*, the only type-II interferon, is a cytokine encoded by the *IFNG* gene and has multiple regulatory roles within both the innate and adaptive immune systems. Interestingly, our data indicated that IFN-*γ* mRNA levels were almost reversed in all of the tested samples among the treatments of CMC-Na, ART, and the combination when compared with WT-sensitive and WT-resistant or corresponding KO groups, respectively. The same changes were also discovered when compared with WT-sensitive and KO-sensitive or WT-resistant and KO-resistant groups. This hinted at the potential involvement of P-gp in IFN-*γ* mRNA expression.

## Conclusions

CHR might play a complex dual role by regulating the crosstalk between ABC-transporter-mediated multidrug resistance and ROS/GSH redox balance-mediated specific resistance. Its upregluation or downregulation occurs simultaneously in different tissues, indicating that CHR might function as a modulator (or homeostasis stabilizer) rather than an inhibitor or scavenger. The PI3K/AKT-mTOR and MAPK signal pathways, as well as IFN-*γ,* may be directly involved in this mechanism. Furthermore, we demonstrated that P-gp exerts direct or indirect regulation on these indexes or pathways.

## Supplementary Information

Supplementary Material 1.

## Data Availability

Data supporting the main conclusions of this study are included in the manuscript.

## Abbreviations

ART: Artemisinin
CHR: Chrysosplenetin
ART–CHR (1:2): Artemisinin and chrysosplenetin in ratio of 1:2
CMC-Na: Sodium carboxymethylcellulose
RIF: Rifampicin
ACTs: Artemisinin-based combination therapies
MN: Sensitive groups without drug treatment (negative control)
MC: Sensitive groups under CMC-Na treatment (negative control)
MA: Sensitive groups under ART treatment
MF: Sensitive groups under the combination treatment
KN: Resistant groups without drug treatment
KC: Resistant groups under CMC-Na treatment
KA: Resistant groups under ART treatment
KF: Resistant groups under the combination treatment
MR: KO-sensitive groups under RIF treatment
KR: KO-resistant groups under RIF treatment
