# Supplementary material for: Chrysosplenetin acts as a homeostasis stabilizer with dual-function in shattering Plasmodium berghei K173 resistance to artemisinin driven by both ABC transporters and heme-ROS/GSH axis
Source: Parasit Vectors. 2025 Oct 9;18:404. doi: 10.1186/s13071-025-07018-0 (PMC12512365; doi:10.1186/s13071-025-07018-0)

Additional file 1. Figure 1. Genotyping-based identification of homozygous KO mice versus WT mice using tail DNA samples.


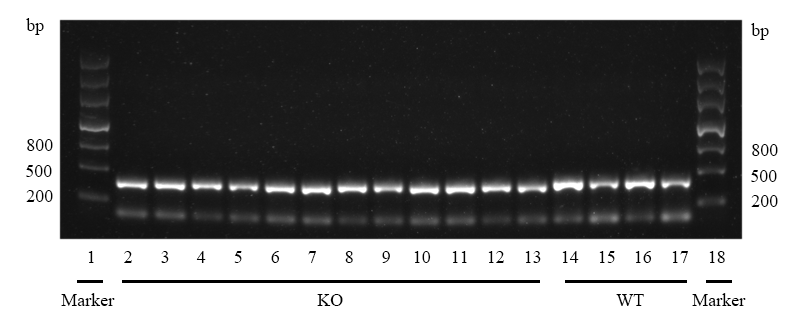


Additional file 2. Figure 2. (*A*) Microscopic analysis of RBCs without parasite-infection by Wright's staining. (*B*) Microscopic analysis of RBCs infection with the sensitive parasite strain by Wright's staining. (*C*) Microscopic analysis of RBCs infection with the resistant parasite strain by Wright's staining.


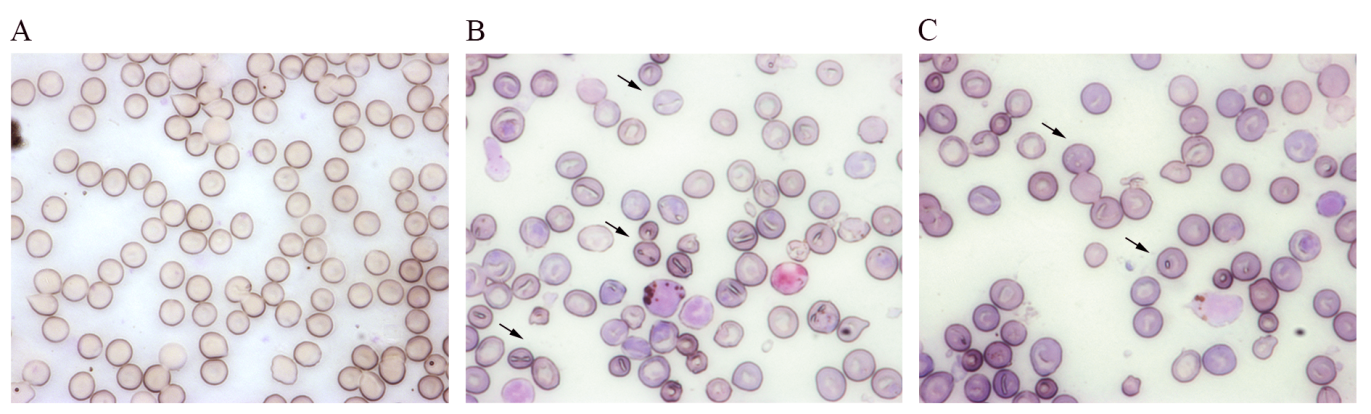


Additional file 3. Figure 3. The ART-CHR combination reshapes spatial and drug resistance-phenotypic disparities in the mRNA expressions of Mrps (Mrp1, 2, 4, and 5) and Bcrp under *Mdr1a* deficiency (*n*=4 mice per group). All data were presented as $\overline{x}$ ± standard deviation (SD). The data were subjected to one-way analysis of variance (ANOVA) to detect significant differences among study groups. The Student-Newman-Keuls (SNK) was applied to determine difference between means with significance levels set at *P*<0.05, *P*<0.01, and *P*<0.001. **i** Comparisons between WT-Sensitive and WT-Resistant subgroups; **ii** Comparisons between KO-Sensitive and KO-Resistant subgroups; **iii** Comparisons between WT-Sensitive and KO-Sensitive subgroups; **iv** Comparisons between WT-Resistant and KO-Resistant subgroups. **A**, **E**, **I**, **M**, **Q** Intestine samples; **B**, **F**, **J**, **N**, **R** Liver samples; **C**, **G**, **K**, **O**, **S** Blood samples; **D**, **H**, **L**, **P**, **T** Spleen samples. WT: Wild-type; KO: Knock out.

CMC-Na: Carboxymethyl cellulose sodium, 13 mL/kg; ART: Artemisinin monotherapy, 40 mg/kg; ART-CHR (1:2): Artemisinin-chysosplenetin combination in ratio of 1:2, 40:80 mg/kg; RIF: Rifampicin, 210 mg/kg. In the bar chart, WT-Sensitive *vs.* WT-Resistant groups are distinguished by white and blue; KO-Sensitive *vs.* KO-Resistant by red and gray; WT-Sensitive *vs.* KO-Sensitive by white and red; and WT-Resistant *vs.* KO-Resistant by blue and gray, ensuring clear differentiation (the same applies below)


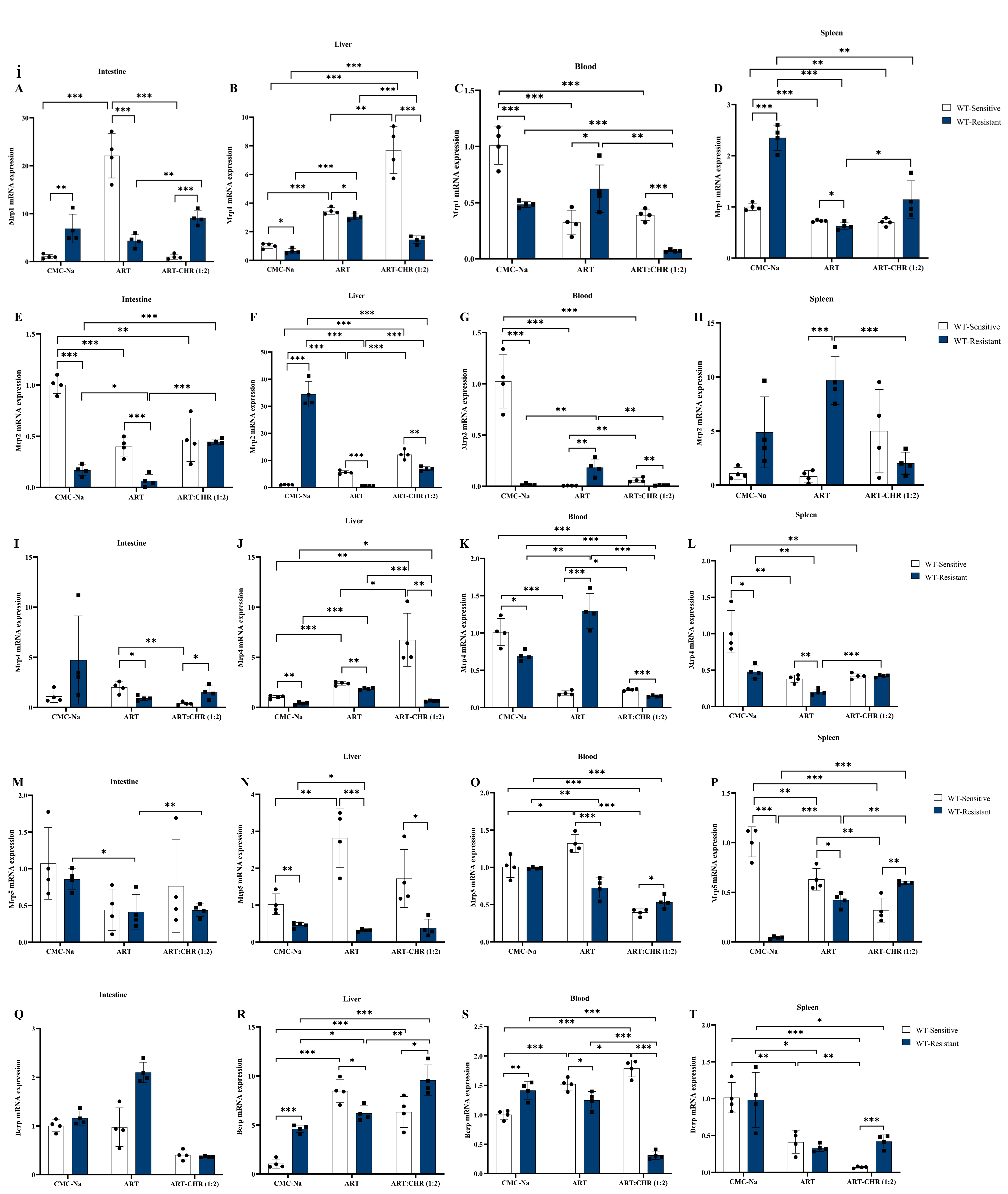


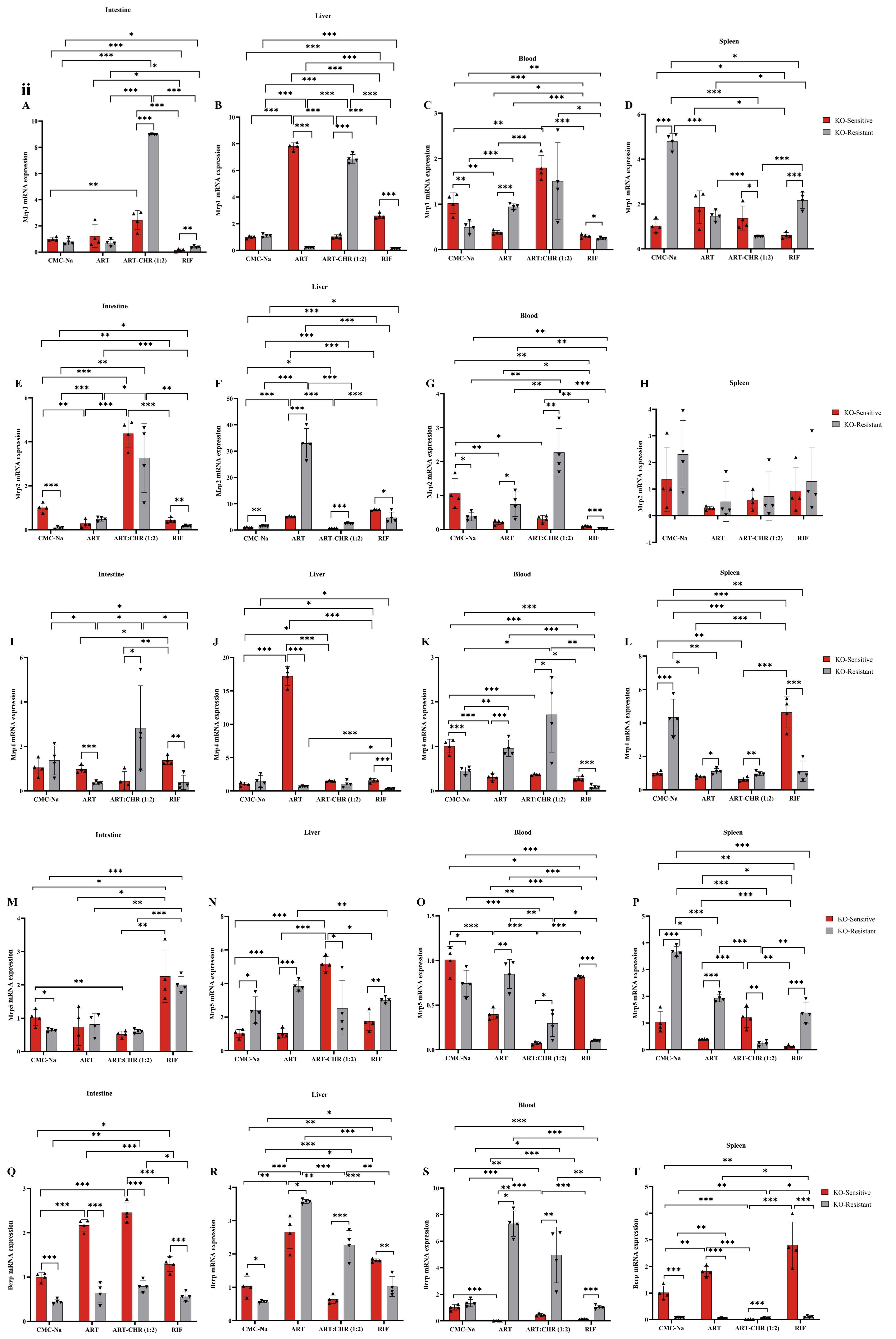


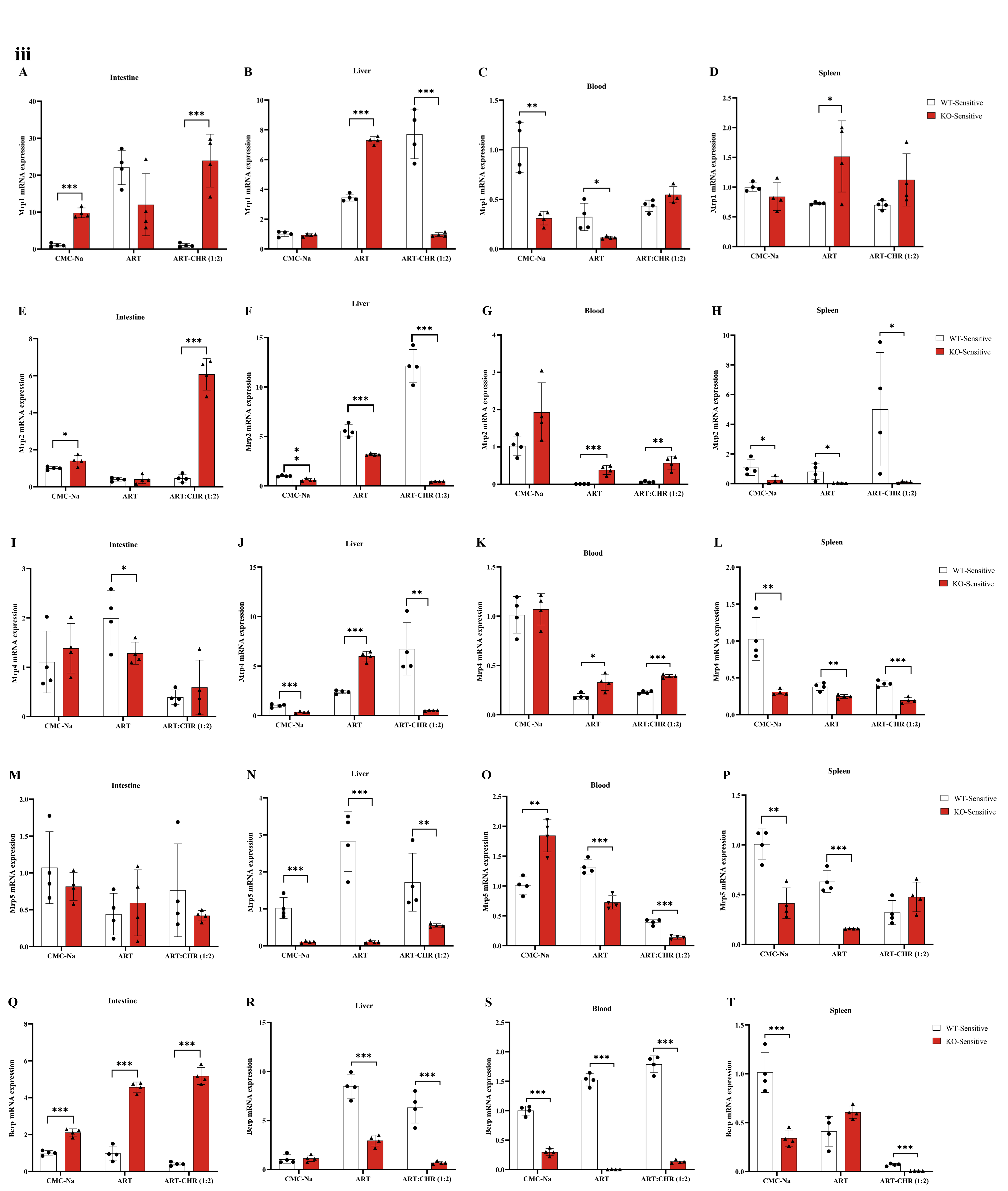


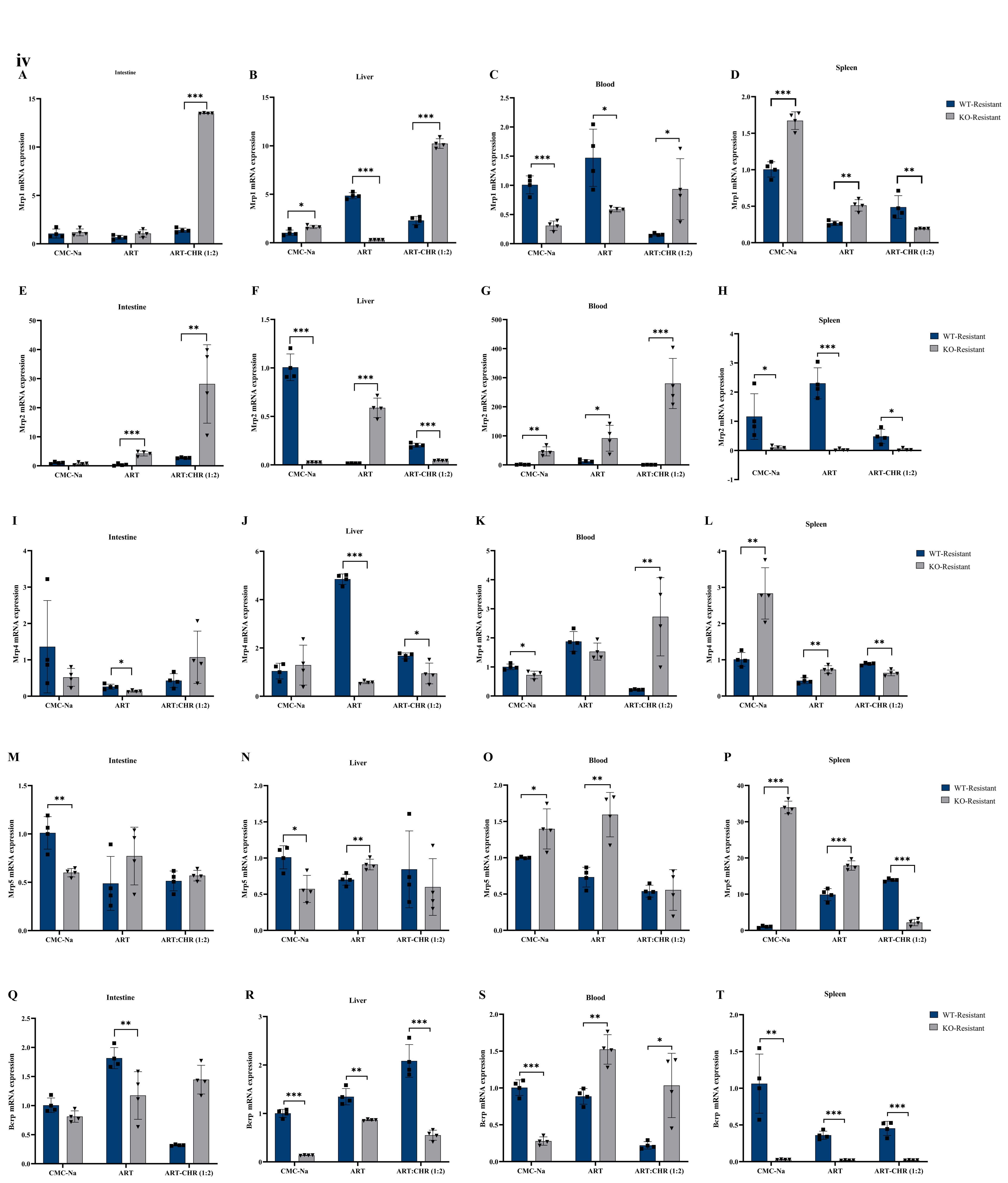


Additional file 4. Figure 4. ART-CHR combination inhibits PI3K/AKT-mTOR signaling pathway dysregulated by *Mdr1a* deficiency (*n*=4 mice per group). All data were presented as $\overline{x}$ ± standard deviation (SD). The data were subjected to one-way analysis of variance (ANOVA) to detect significant differences among study groups. The Student-Newman-Keuls (SNK) was applied to determine difference between means with significance levels set at *P*<0.05, *P*<0.01, and *P*<0.001. **i** PI3K mRNA expressions detected by RT-qPCR; **ii** AKT mRNA expressions detected by RT-qPCR; **iii** mTOR mRNA expressions detected by RT-qPCR. **A**, **E**, **I**, **M**, **Q** Intestine samples; **B**, **F**, **J**, **N**, **R** Liver samples; **C**, **G**, **K**, **O**, **S** Blood samples; **D**, **H**, **L**, **P**, **T** Spleen samples. MN: sensitive groups without drug treatment (negative control); MC: sensitive groups under CMC-Na treatment (negative control); MA: sensitive groups under ART treatment; MF: sensitive groups under the combination treatment; KN: resistant groups without drug treatment; KC: resistant groups under CMC-Na treatment; KA: resistant groups under ART treatment; KF: resistant groups under the combination treatment; MR: KO-Sensitive groups under RIF treatment; KR: KO-Resistant groups under RIF treatment


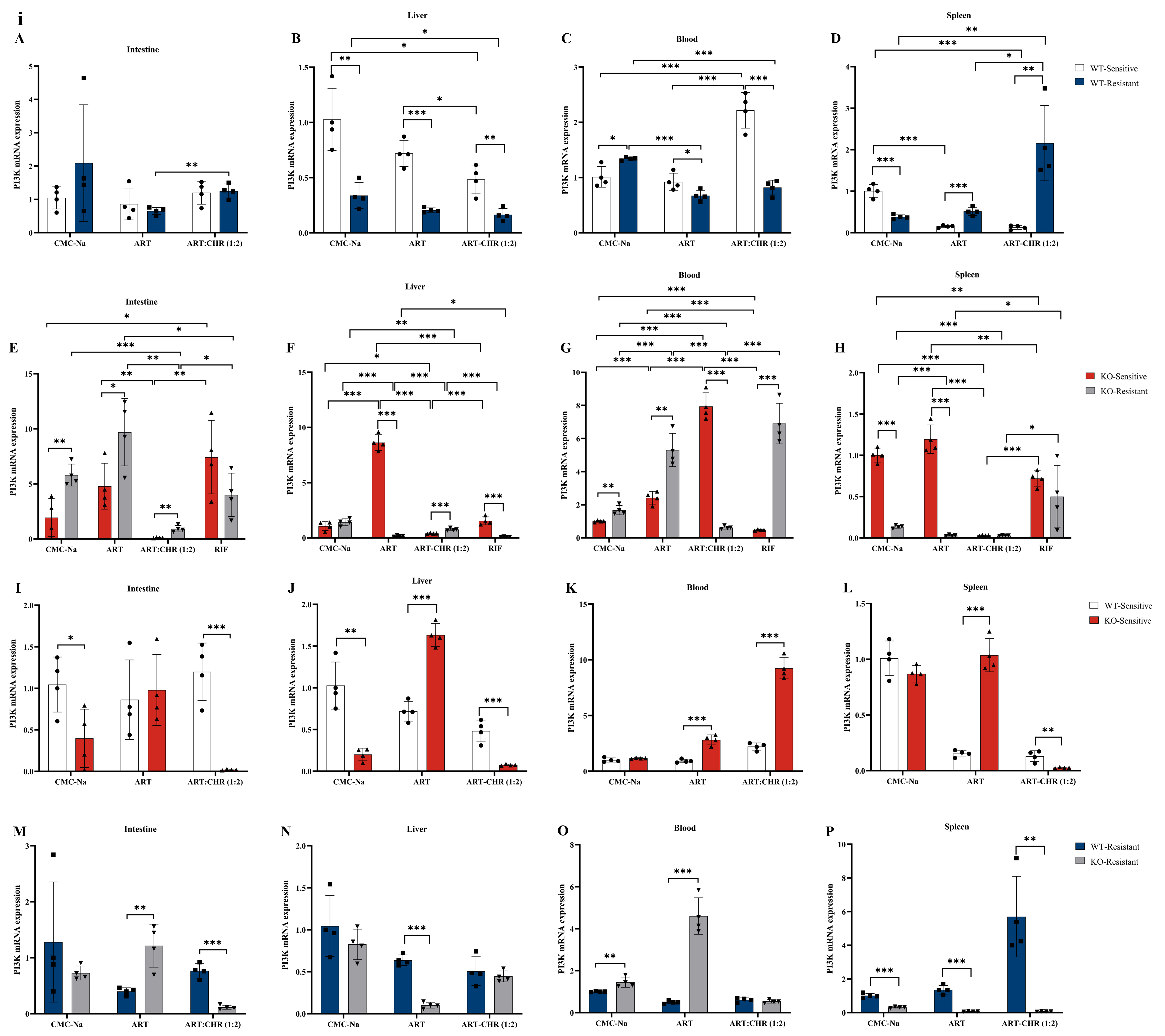


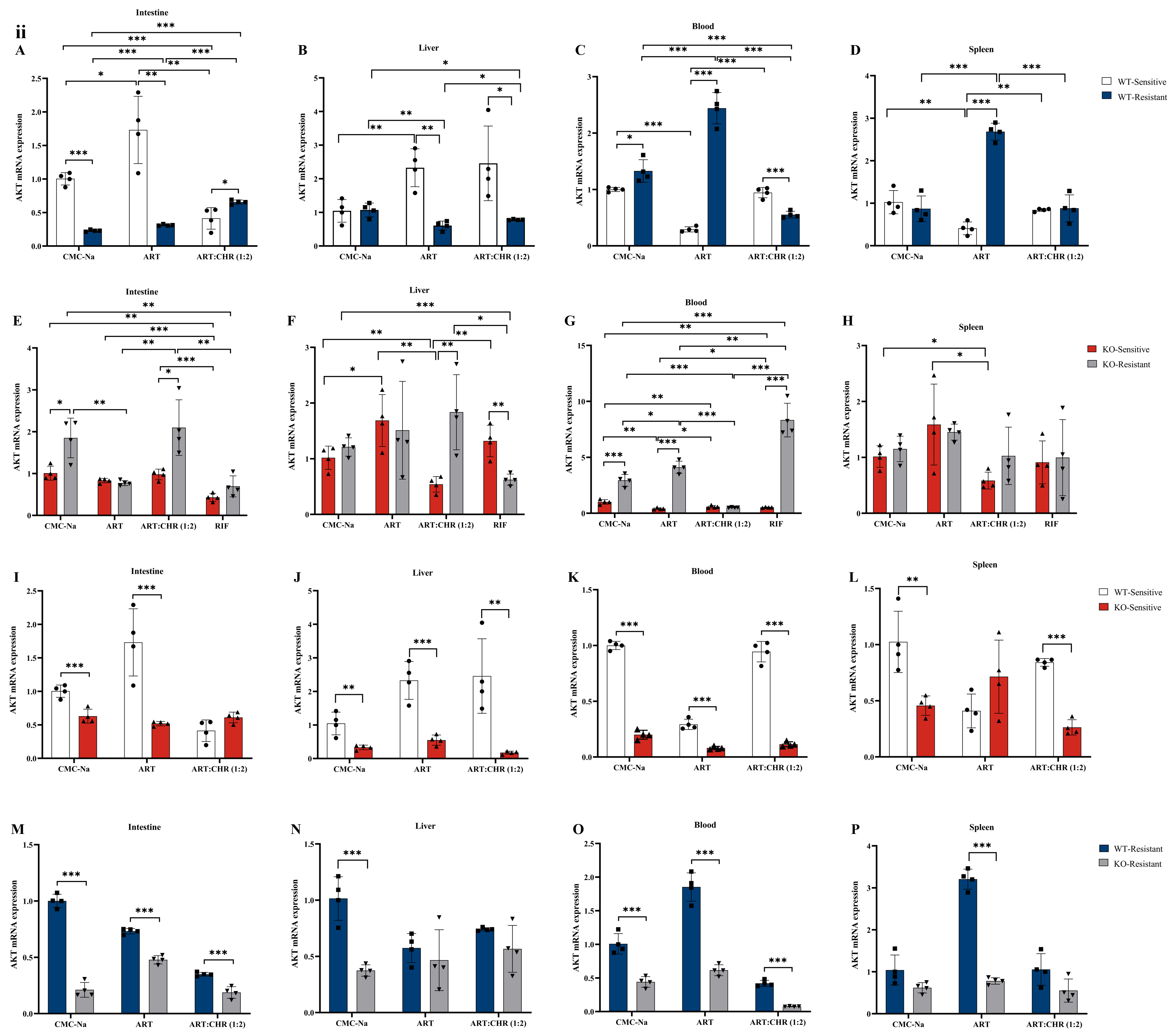


**
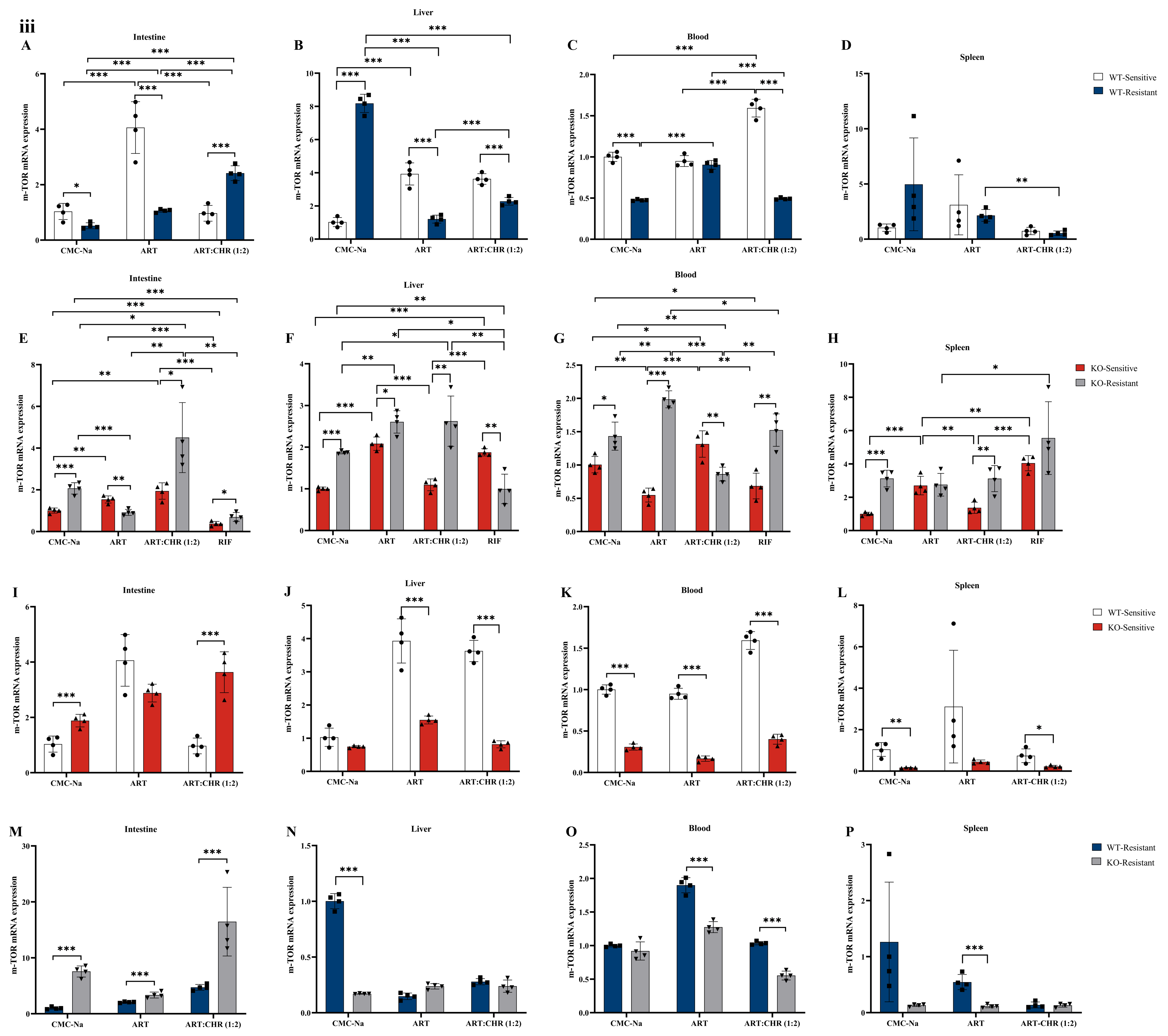
**

Additional file 5. Figure 5. P-gp-independent spatially antagonistic regulation of ART alone and ART-CHR combination therapy on MAPK pathway (*n*=4 mice per group). All data were presented as $\overline{x}$ ± standard deviation (SD). The data were subjected to one-way analysis of variance (ANOVA) to detect significant differences among study groups. The Student-Newman-Keuls (SNK) was applied to determine difference between means with significance levels set at *P*<0.05, *P*<0.01, and *P*<0.001. **i** ERK mRNA expression levels; **ii** JNK mRNA expression levels; **iii** P38 mRNA expression levels. **A**, **E**, **I**, **M**, **Q** Intestine samples; **B**, **F**, **J**, **N**, **R** Liver samples; **C**, **G**, **K**, **O**, **S** Blood samples; **D**, **H**, **L**, **P**, **T** Spleen samples


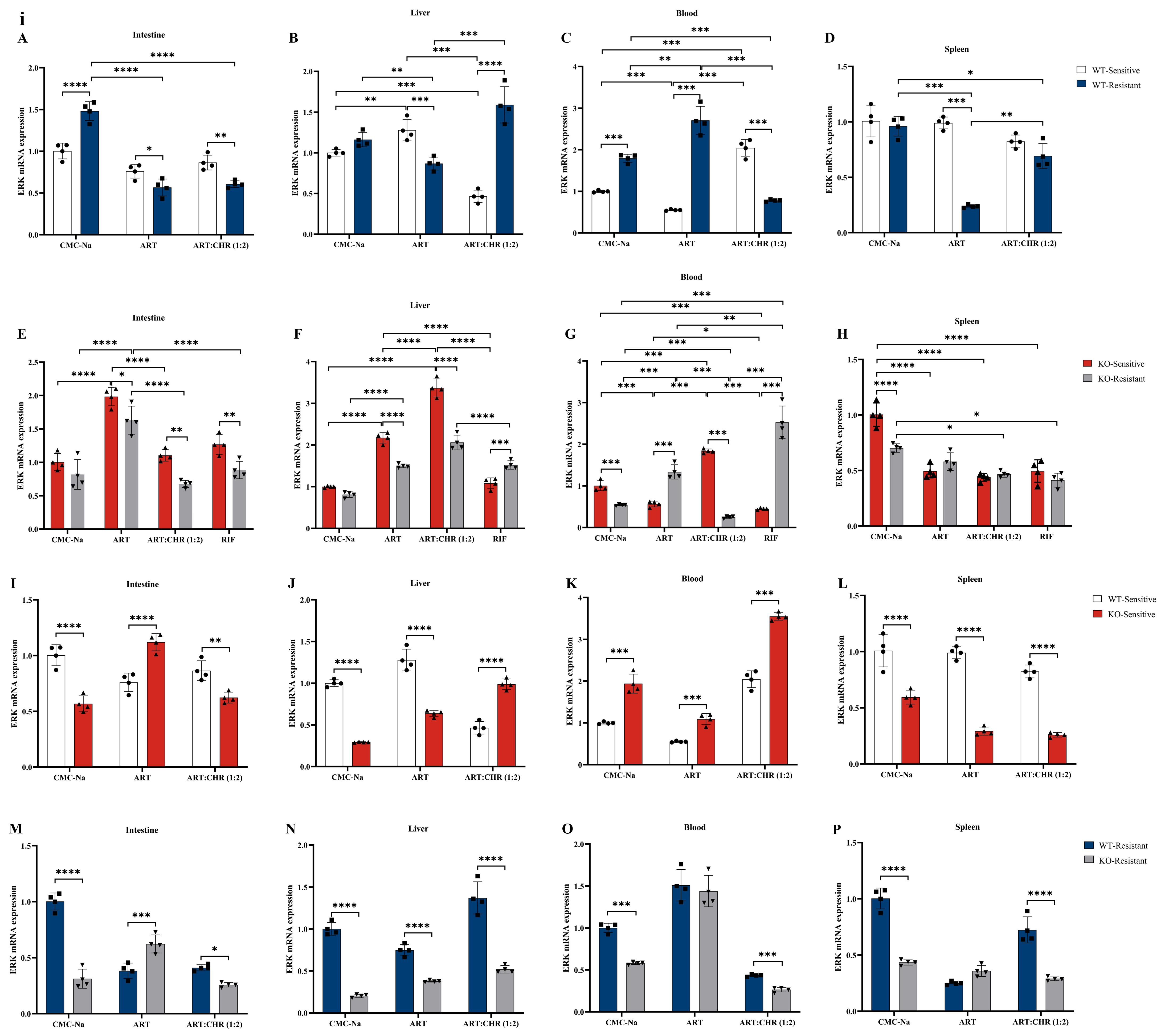


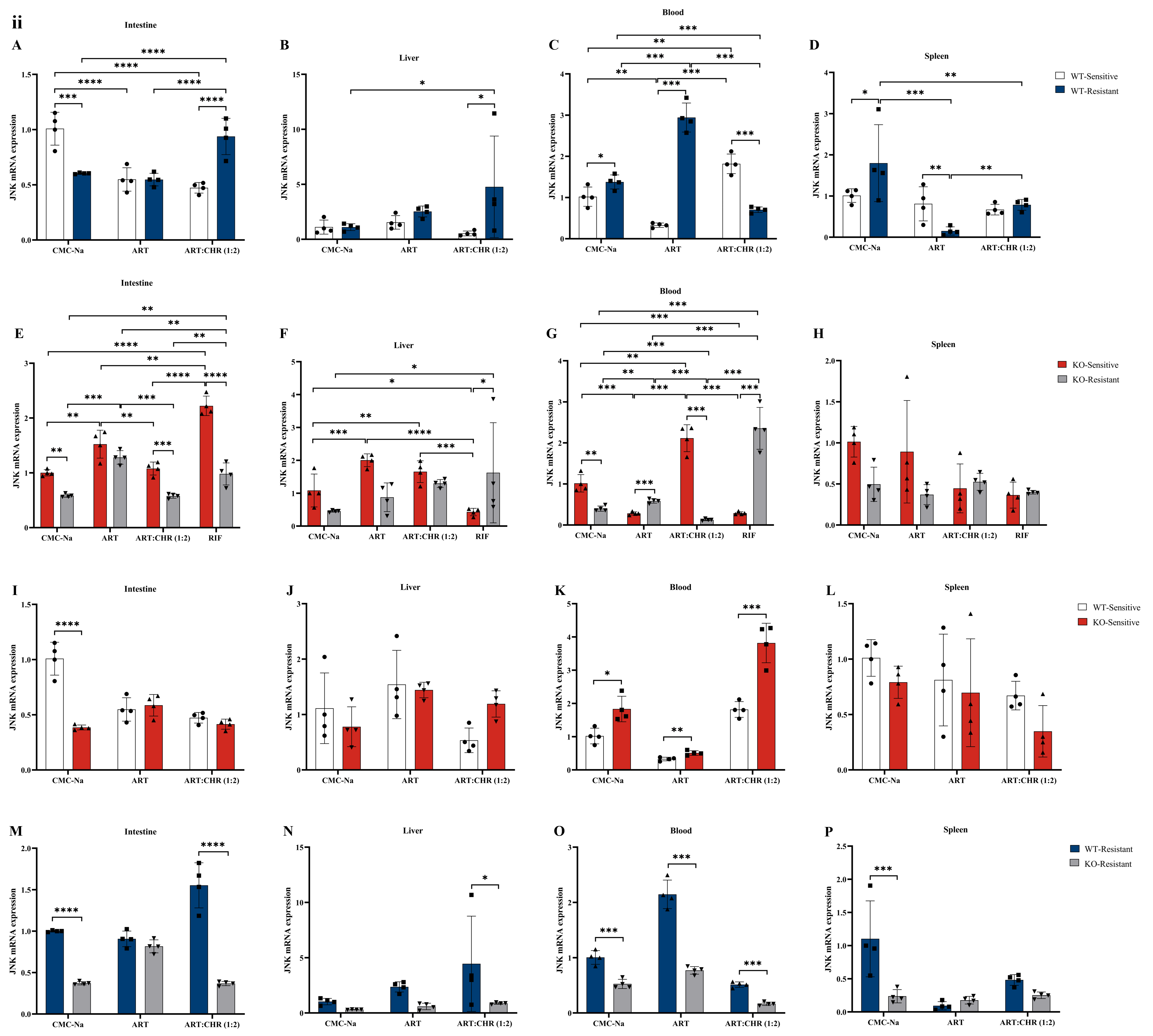


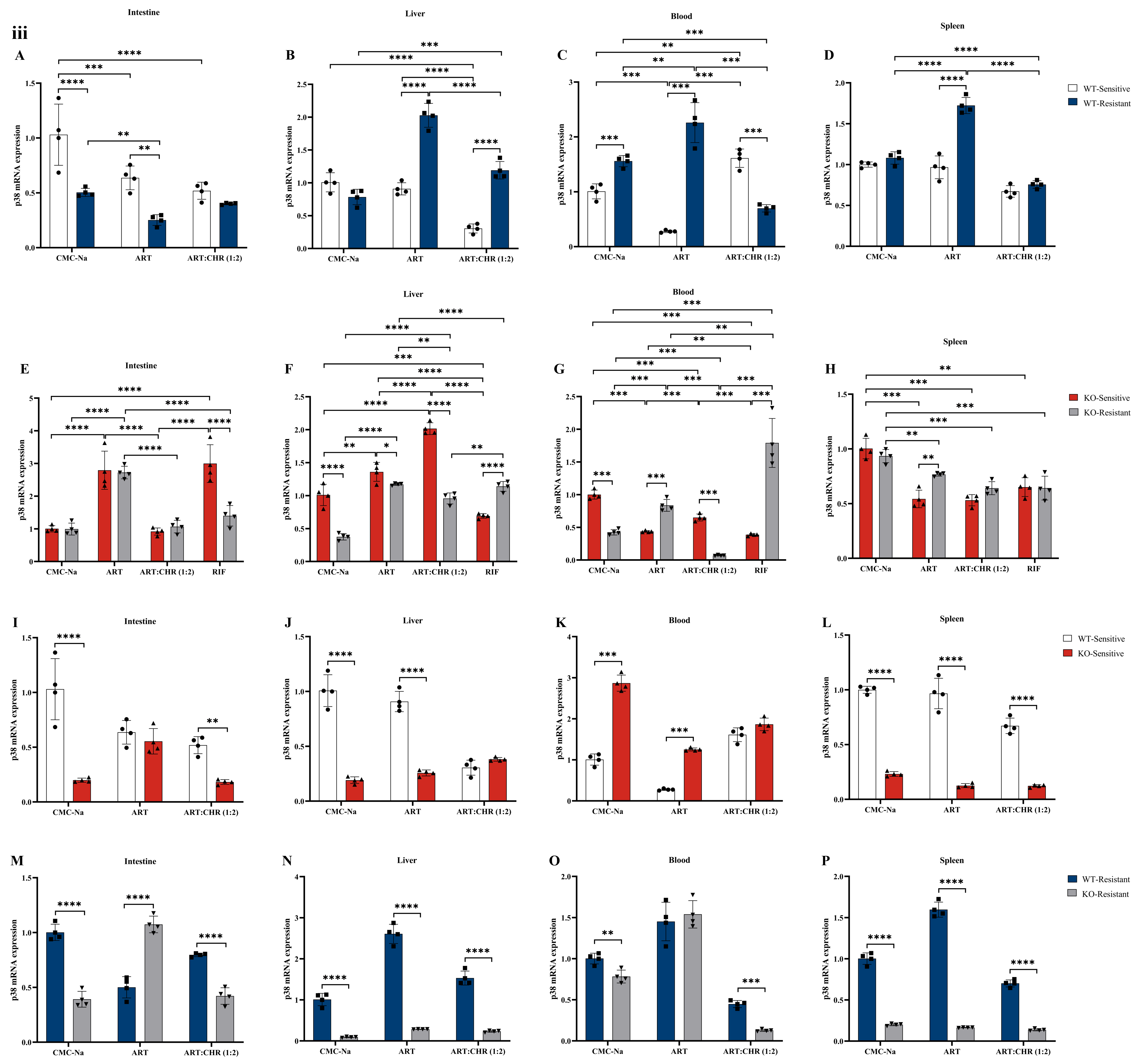


Additional file 6. Figure 6. Opposing regulation of cytokine expressions by ART monotherapy and ART-CHR combination in a spatial-, parasite phenotype-, and P-gp-dependent manner (*n*=4 mice per group). All data were presented as $\overline{x}$ ± standard deviation (SD). The data were subjected to one-way analysis of variance (ANOVA) to detect significant differences among study groups. The Student-Newman-Keuls (SNK) was applied to determine difference between means with significance levels set at *P*<0.05, *P*<0.01, and *P*<0.001. **i** IFN-*γ* mRNA levels; **ii** IL-1*β* mRNA levels; **iii** TNF-*α* mRNA levels. **A**, **E**, **I**, **M**, **Q** Intestine samples; **B**, **F**, **J**, **N**, **R** Liver samples; **C**, **G**, **K**, **O**, **S** Blood samples; **D**, **H**, **L**, **P**, **T** Spleen samples


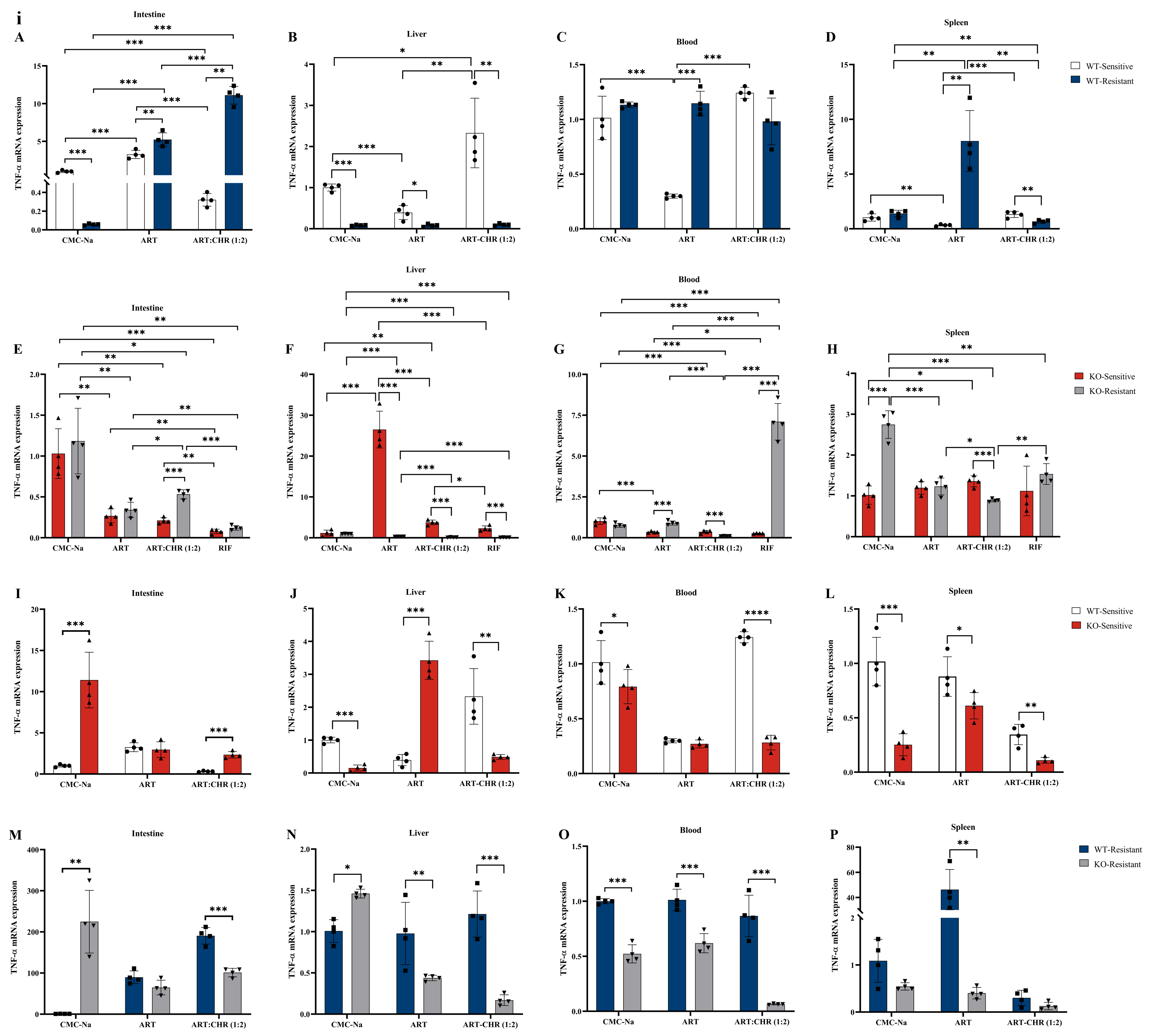


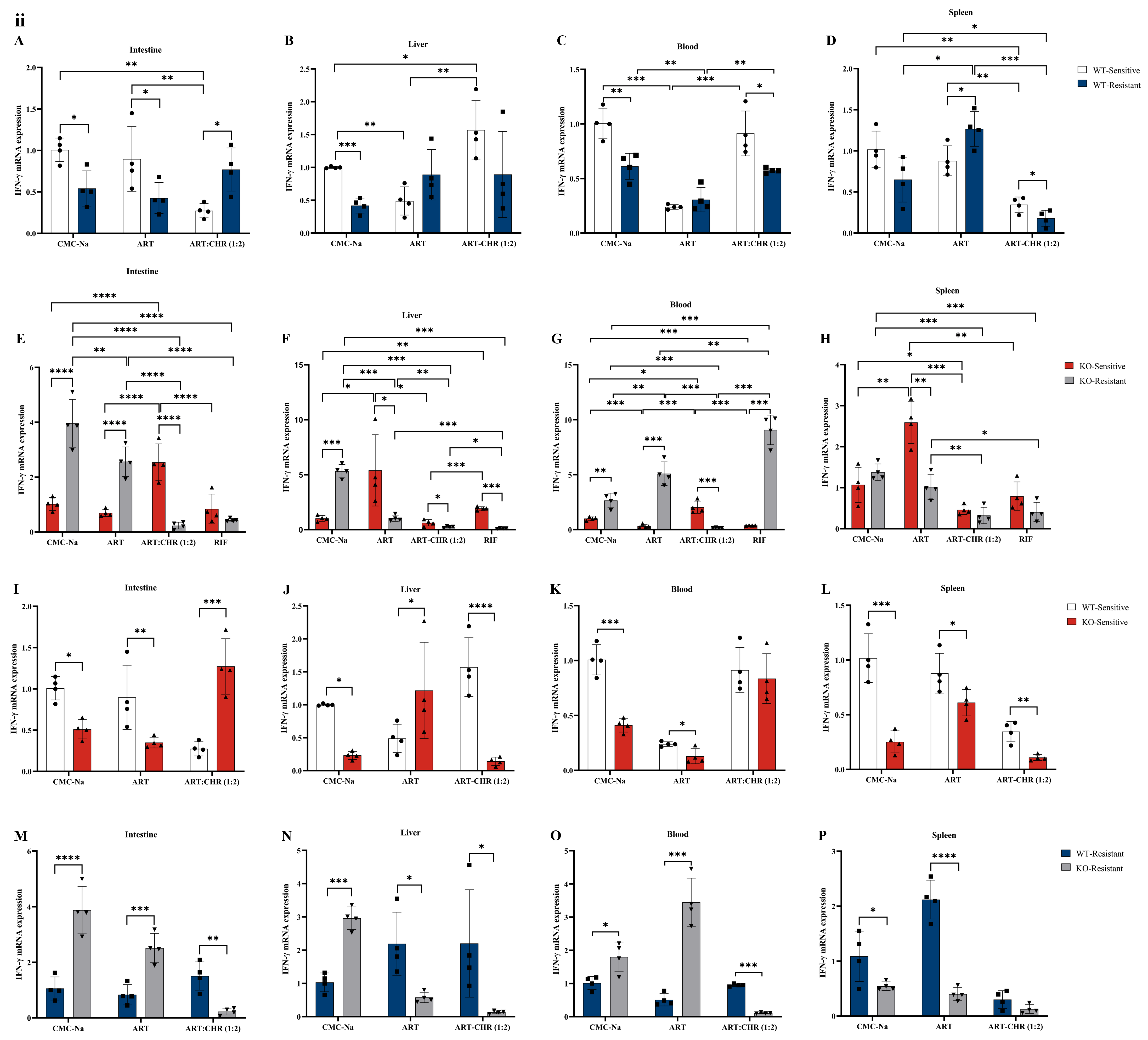


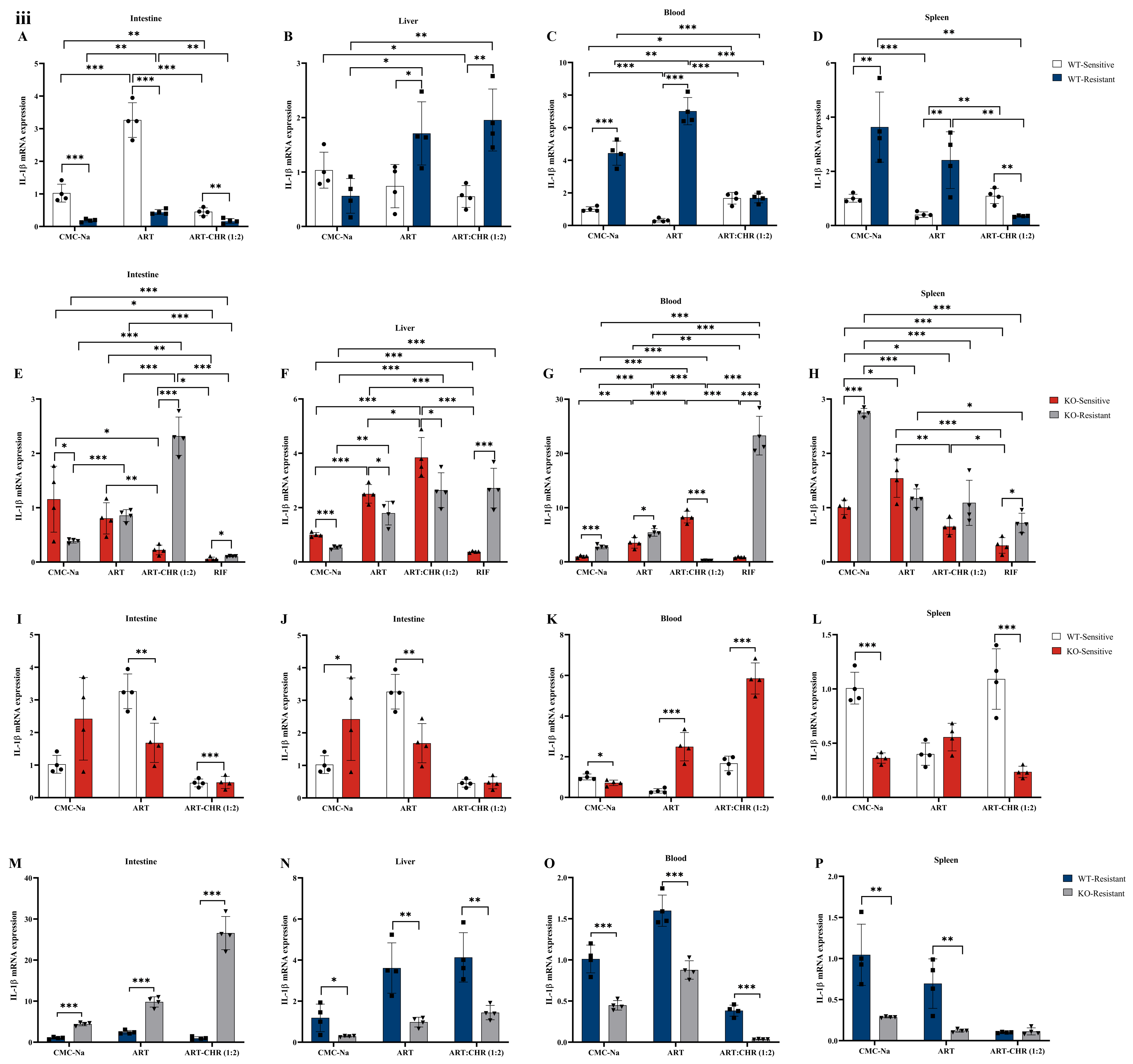

Supplement: Supplementary file 1 — Supplementary Material 1. [file 13071_2025_7018_MOESM1_ESM.doc]
